# Unraveling the role of satellite DNAs in the evolution of the giant XY sex chromosomes of the flea beetle *Omophoita octoguttata* (Coleoptera, Chrysomelidae)

**DOI:** 10.1186/s12915-025-02155-5

**Published:** 2025-02-21

**Authors:** Jhon Alex Dziechciarz Vidal, Deborah Charlesworth, Ricardo Utsunomia, Manuel A. Garrido-Ramos, Rodrigo Zeni dos Santos, Fábio Porto-Foresti, Roberto Ferreira Artoni, Thomas Liehr, Mara Cristina de Almeida, Marcelo de Bello Cioffi

**Affiliations:** 1 Laboratory of Evolutionary Cytogenetics, Department of Genetics and Evolution, Federal University of São Carlos, São Carlos, SP Brazil; 2 Institute of Ecology and Evolution, University of Edinburgh, Edinburgh, UK; 3 Faculdade de Ciências, UNESP, Bauru, São Paulo, Brazil; 4 Departamento de Genética, Facultad de Ciencias, Universidad de Granada, Granada, 18071 Spain; 5 Laboratory of Genetics and Evolution, Department of Molecular Structural Biology and Genetics, State University of Ponta Grossa (UEPG), Av. Carlos Cavalcanti, Ponta Grossa, 4748 Brazil; 6 Institute of Human Genetics, Jena University Hospital, Friedrich Schiller University, Jena, Germany

**Keywords:** Repetitive DNA, Satellitome, Transposable elements

## Abstract

**Background:**

The flea beetle *Omophoita octoguttata* (Coleoptera, Chrysomelidae) is a member of a group in which the males completely lack meiotic recombination (male-specific achiasmy) and that have extraordinarily large X and Y chromosomes. We combined genome sequencing, including microdissected Y and X chromosomes, and cytogenetic in situ hybridization studies, to evaluate the potential role of satellite DNAs (satDNAs) in the differentiation of those gigantic sex chromosomes.

**Results:**

We report flow cytometry results showing that this species has a very large genome size (estimated to be 4.61 and 5.47 pg, or roughly 4.6 and 5.5 gigabases, for males and females, respectively), higher than the estimates from two other Alticinae species without giant sex chromosomes, suggesting that these sequences have greatly expanded on both the sex chromosomes, and that the Y has not greatly shrunk like the ones of other insects such as *Drosophila* with male achiasmy. About 68% of this large genome is made up of repetitive DNAs. Satellite DNAs (OocSatDNAs) form ~ 8–9% of their genomes, and we estimate how much of the sex chromosome expansions occurred due to differential amplification of different satellite classes. Analysis of divergence between sequences in the X and Y chromosomes suggests that, during the past roughly 20 mya, different OocSatDNAs amplified independently, leading to different representations. Some are specific to the Y or X chromosome, as expected when males are achiasmate, completely preventing genetic exchanges between the Y and X.

**Supplementary Information:**

The online version contains supplementary material available at 10.1186/s12915-025-02155-5.

## Background

Sex chromosomes are thought to evolve after a sex-determining gene or genes evolve on a chromosome and recombination becomes suppressed [1–5]. The absence of recombination results in the initially similar proto-Y/W chromosome pair differentiating, as the non-recombining member of the pair accumulates sequence differences and repetitive DNAs [6], which can lead to an initial increase in size (reviewed in [7]). Large sex chromosomes have indeed been reported in several animal and plant groups [8–12]. Repetitive sequence accumulation in Y chromosomes of XY systems and W chromosomes of ZW species may involve transposable elements (TEs) (e.g., [13]) and/or satellite DNAs (satDNAs) [14]. Later, rearrangements may occur, and genetic degeneration (loss of gene functions or deletions of genes) may eventually create strongly heteromorphic sex chromosome pairs, usually with the non-recombining member of the pair smaller than the recombining one (reviewed in [15]). In taxa with old-established sex chromosomes, like mammals, the Y is indeed generally smaller than the X (reviewed in [16]), and in birds, the W is often smaller than the Z, though the relative sizes vary in both these groups [17], and sometimes one or both members of the pair are enlarged by sex chromosome-autosome fusions. In *Drosophila*, for example, the Y is often small, except after a fusion with an autosome has created a neo-sex chromosome too recently for genetic degeneration to have occurred due to the complete lack of meiotic recombination in males, allowing major deletions to occur (e.g., [18]).

Three cases are known of giant sex chromosomes that probably do not involve fusions. One example is the large X in rodents of the mammalian genus *Microtus* [19–23]. Another case is in *Drosophila pallidipennis* [24], whose X and Y sizes are each equal to about the sum of the lengths of the four large autosomes, or roughly half of the total genome size; this species has not been studied further. The third case, in beetles (Coleoptera), is equally striking particularly in *Oedionychina* species in the Chrysomelidae [25–29], native to the Neotropics [30]. In this group of flea beetles, both sex chromosomes are regularly at least 10 times the size of the largest autosomes [25, 31].

*Oedionychina* (*Oedionychini* sensu Chapuis, 1875) karyotypes are stable, with a diploid chromosome number of 2n = 22 = 10II + X + Y [25]. All species have acrocentric autosomes [25], and giant X and Y chromosomes, which are always metacentric [25–29, 32]. Phylogenetic studies suggested that the genus *Omophoita* diverged in the mid-Cretaceous [33]. Large X and Y chromosomes therefore probably initially evolved about 100 million years ago (my). They are shared with other *Oedionychina* species, and only a few species lack giant X chromosomes (for example, *Asphaera*, which have multiple sex chromosomes, thought to result from fissions) [34]. Whole genome painting indicates that the X is extensively conserved in related species [31].

As the number of autosomes is only one less than the most common diploid number of Chrysomelidae (*n* = 12) [35], the giant X and Y chromosomes cannot be explained only by the occurrence of multiple sex chromosome-autosome fusions. Huge accumulations of repetitive DNA sequences in large heterochromatic regions are probably the primary cause of the sex chromosome enlargement in both *Microtus* [21–23] and *Oedionychina* [25, 36]. In *Oedionychina*, Rosolen et al. [37] suggested TE accumulation. Asynapsis, ensuring the complete absence of recombination, almost invariably occurs in the heterogametic sex and has evolved independently at least 26 times (reviewed in [38]). It is widespread in male Diptera such as *Drosophila* [39] and is documented in male *Omophoita* [26]. Accumulation of repetitive sequences is therefore plausible in *Omophoita* Y chromosomes, which are confined to males.

Repetitive DNAs, particularly TEs, and satDNAs are the most abundant sequences of most eukaryotic genomes [6, 40, 41], and are important contributors to genome architecture, and specifically show consistent enrichment in regions where recombination is infrequent [42]. The recent availability of low-coverage sequencing and bioinformatics tools for analyses [43], even in non-model organisms without reference genomes, has allowed the “satellitomes” (the entire set of satDNAs in a species’ genome) to be studied in a variety of organisms [44, 45]. SatDNAs are generally strongly concentrated in regions near the centromeres of chromosomes [6, 40], and genome sequencing allows their locations to be studied in detail, including differences between closely related species that were previously known only through cytogenetic studies [46]. These studies have confirmed that satellites tend to be concentrated in chromatin that is distinct from euchromatin, often in highly gene-poor heterochromatin [47], although high-throughput genome sequencing has revealed the existence of additional short arrays of repeat units scattered throughout the genome in euchromatin [44, 45, 48, 49]. The same satDNA sequence, or the total amount of such sequences, can be present in very different amounts between populations and related species [50–53], with sometimes as much as a twofold difference in genome size, as in species of the hemipteran bug *Triatoma*, where at least half of the *T. delpontei* genome consists of such sequences, mainly in heterochromatin [50]. As sequencing heterochromatic regions is still very difficult, most sequence assemblies still concentrate on euchromatic regions. Mapping satDNAs to their chromosomal locations [14, 54–57] therefore remains very valuable, even though their abundances in different genome regions cannot be precisely quantified, which will be needed for a full understanding of how they evolve.

In *Omophoita octoguttata*, the X is even larger than the Y; the average lengths, based on mitotic chromosome sizes (which are rough, as they are affected by differences in condensation between cells), are 80 μm for the X, slightly larger than the 70 μm estimate for the Y. The autosomes range from 5 to 7 µm, making the largest autosome about 10 times smaller than the X or Y [25]. As outlined in the Discussion section below, while Y chromosomes are predicted to expand during their evolution, X chromosomes are not. Here, by combining genome sequencing, including microdissected Y and X chromosomes, and cytogenetic in situ hybridization analyses, we evaluate the contributions of satDNAs to the evolution of these gigantic sex chromosomes, including the surprisingly large X.

## Results

### The *O. octoguttata* genome size and sizes of the sex chromosomes

The *O. octoguttata* diploid genome size is estimated to be about 4.61 picograms (pg) based on 5 males (about 4.6 gigabases, Gb) and 5.47 pg (about 5.5 Gb) based on 6 females (Fig. S1). This is higher than estimates from other Alticinae species without giant sex chromosomes (0.482 pg or about 400 Mb in *Crepidodera plutus* (though *Podagrica fuscicornis* also has a very large genome size of 3.956 pg or about 4 Gb)) [58]. The approximate size of the X can be calculated from the information that it is about 10 times larger than the largest of the 11 autosomes. Assuming, conservatively, that all autosomes are one-tenth of the X size, a total haploid female genome size of 2.75 Gb then implies that the autosomes are roughly 130 Mb each, and the X must be about 1.3 Gb, an astonishing amount for a single chromosome. The 900 Mb larger genome size in our sample of females, compared with the males, is consistent with the size differences seen in the karyotype.

### Repeat composition of the *O. octoguttata* genome

To quantify the major repetitive families, we analyzed RepeatMasked sequences by graph-based clustering (see Methods). About 68% of the *O. octoguttata* genome consists of repetitive sequences (68.54 in males and 67.66 in females). In both sexes, the largest correctly annotated contributors to the species’ highly repetitive DNA are LTR/gypsy elements (~ 13%) and satellite DNAs (~ 8–9%) (Fig. S2). Most sequences in the clusters we detected are represented similarly in the genomes of individuals from both sexes, but three specific satDNAs (named OocSat15, OocSat20, and OocSat21) showed M/F ratios > 1, sometimes greatly so, indicating differences in abundance between the sexes (Fig. S3; Tables S1 and S2). FISH experiments described below confirm that, as expected, these satDNAs are also highly represented on the Y chromosome.

### In silico analysis of the *Omophoita octoguttata* satellitome

The satellitome of *O. octoguttata* includes 49 satDNAs (Table S1). Homology analysis identified 3 superfamilies using the entire OocSatDNA set, with within-superfamily identity values of ~ 50–70%, 57%, and 53%; we named these SF-1, SF-2, and SF-3, respectively. Forty OocSatDNAs are long tandem repeats of unit sequences whose lengths (RULs) range from 16 to 5042 bp (22 have RULs longer than 1 kb). Their A + T content ranges from 46 to 75% (Table S1).

All 49 OocSatDNAs were found in both sexes, but in different abundances (Table S1; Fig. S4). OocSat21 was 114 times as abundant in the genome of males than females, suggesting that the Y chromosome carries many of these repeats, and 6 others differed to a lesser extent. Six OocSats had significantly higher abundance in the female than the male genome, with a F/M ratio of 4.7 for OocSat48 and 1.7 for OocSat09 (Table S1; Fig. S4). Because males also carry an X chromosome, high F/M ratios can arise only if the X has much higher abundance than the Y. These results are therefore conservative in suggesting both Y- and X-specific accumulation of individual OocSatDNAs. We tested this further by estimating the abundance of OocSatDNA from separate libraries made from microdissected X and Y chromosomes, which confirmed different abundances of individual satDNAs in the two sex chromosomes (Table S2).

### Chromosomal mapping of OocSatDNAs and telomeric repeats

**Fig. 1:**
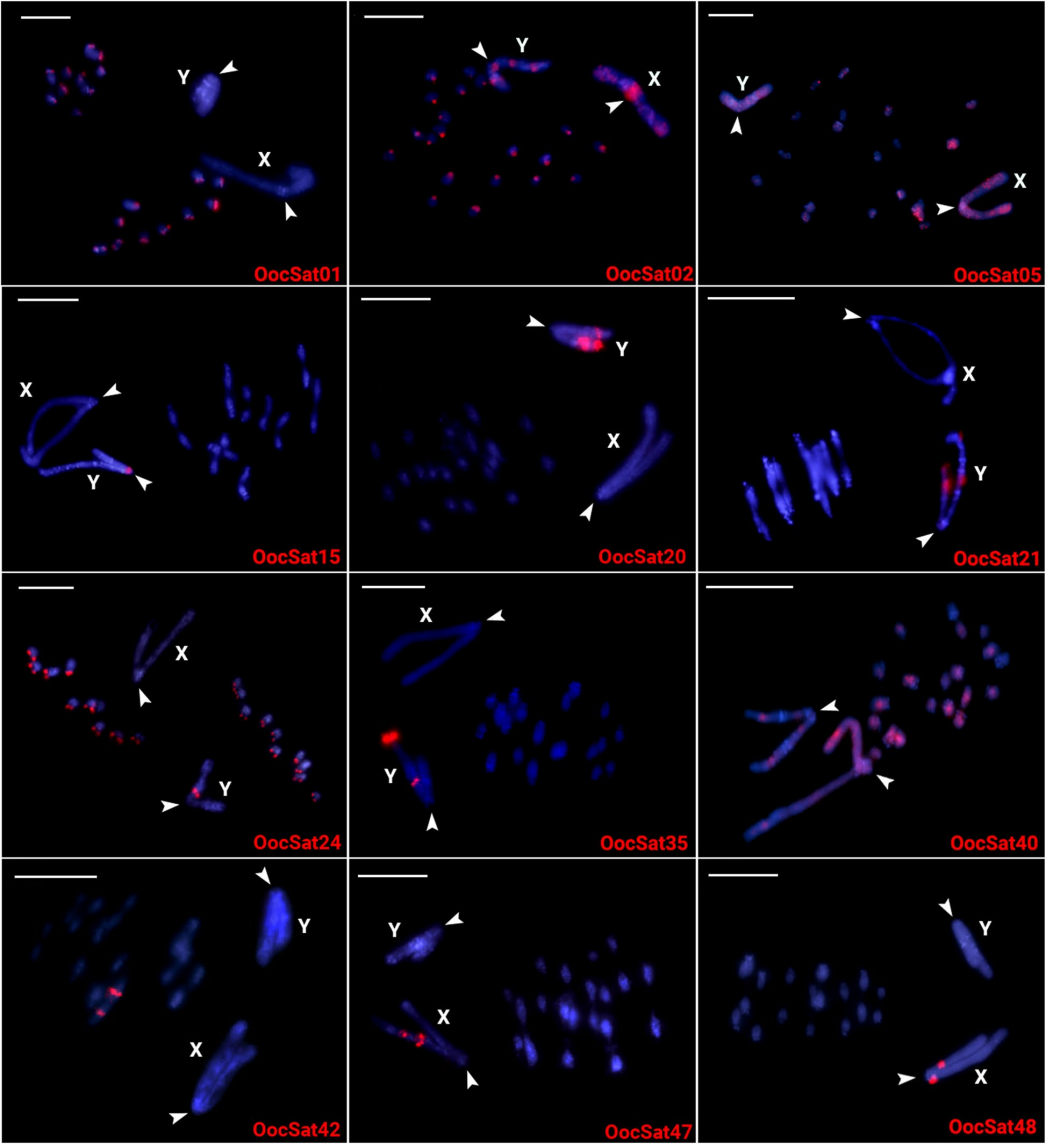
*O. octoguttata* chromosomes in male meiosis, showing the locations of OocSatDNAs (red signals—ATTO550 labeled). Letters indicate the X and Y chromosomes and arrowheads indicate their centromeric regions. Bars = 20 µm

**Fig. 2:**
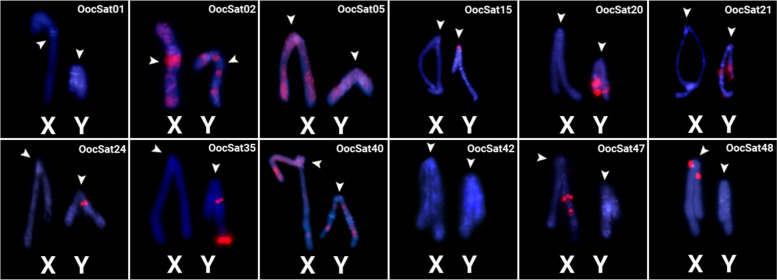
Images extracted from the same preparations shown in Fig. 3 showing the locations of OocSatDNAs on the *O. octoguttata* X and Y chromosomes. Letters indicate the X and Y chromosomes and arrowheads indicate the centromeric heterochromatic regions

Eleven OocSatDNAs were studied by FISH experiments (Figs. 1 and 2). Table S3 summarizes the findings, which reveal pronounced sex chromosome-specific patterns for some of the OocSatDNAs studied, as would be expected given the lack of crossing over between the X and Y in this species. Two OocSatDNAs show no evidence of accumulation on the sex chromosomes: OocSat01 was found in the centromeric regions of all autosomes but not the sex chromosomes and OocSat42 hybridized to only one autosomal pair. For three other OocSatDNAs (02, 05, and 24), both the autosomes and sex chromosomes showed signals: OocSat02 was detected in the centromeric regions of all chromosomes, with extra blocks in non-centromeric regions of both the X and Y (Figs. 1 and 2; Table S3). OocSat05 and OocSat40 signals were scattered across all chromosomes, including the X and Y. Most of the other satDNAs exhibited signals on several chromosomes of *O. octoguttata* (Fig. 1).

**Fig. 3:**
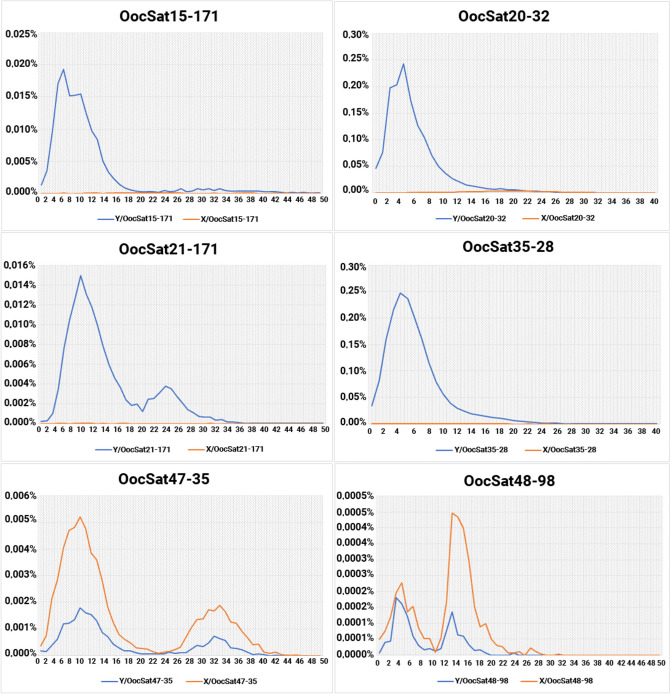
Repeat landscape (RL) plots for six OocSatDNAs. For each satDNA family, the y axes show the estimated abundances of different repeat variants within the family (note the different y axis scales for the different families), and the x axes show the estimated sequence divergence from the consensus sequence (as percentage values), across all site types in the alignments, with correction for saturation using Kimura’s 2-parameter method, as implemented in the software used for the analyses (see the Methods section). The four families shown in the top two rows were found almost exclusively in the Y chromosome sequences, and their RLs suggest recently increased abundances on the Y chromosome, shown by the blue lines with peaks at low sequence divergence values (abundances on the X chromosome, shown by the yellow lines, are very low). In contrast, the two much less abundant families shown in the bottom row, OocSat47-35 and OocSat48-98, have amplified differentially on the X chromosome, as discussed in the text.

Only a few satellites show pronounced differences between the two sex chromosomes. OocSat-02 is present on both the X and Y, but especially the X, with a strong signal near its centromere (Figs. 1 and 2). OocSat24 showed signals in all autosomes but also in a pericentromeric block in the long arms of the Y chromosome (Figs. 1 and 2). Six other OocSatDNAs were detected either only on the X (OocSat47 and OocSat48) or only on the Y (OocSat15, OocSat20, OocSat21, and OocSat35) (Figs. 1 and 2; Table S3).

The telomeric probe displayed the expected signals in the terminal region of all chromosomes. However, an ITS (interstitial telomeric site) was detected on the long arms of the X chromosome (Fig. S5).

### Evolutionary analysis of satDNAs

The TAREAN (TAndem REpeat Analyzer) software [43] generates a consensus monomer sequence for each satDNA cluster, each of which includes thousands of repeats of a specific satellite sequence; individual repeats show some divergence from the consensus and are referred to as sequence (or repeat) variants. Figure 3 shows repeat landscape (RL) plots displaying, for each satDNA family, the divergence of variants from the family’s consensus sequence, together with their estimated abundances in X and Y chromosomes isolated by microdissection (see Methods). Peaks in the plots represent the largest sets of repeats that have similar divergence values, roughly reflecting times of satDNA expansions within a genome [59]. The RLs for each satDNA in males and females indicate major amplification events for all satDNAs at similar times, as expected if most satDNAs are present on the autosomes as well as the sex chromosomes, and amplifications of autosomal arrays often dominate these plots. The sizes of the peaks differ between the sexes, consistent with the Y chromosome undergoing independent amplifications from those on the X, and the RLs of the two sex chromosomes indeed differ (Figs. 3 and S6). These figures show the RL plots for satDNAs that our FISH results (see above) suggest are specific to the Y chromosome (OocSat15-171, OocSat20-32, OocSat21-171, and OocSat35-28) or the X chromosome (OocSat47-35 and OocSat48-98). The RLs show that these satDNAs can have peaks in abundance of sequences with divergence up to about 40% from their consensus sequences, indicating that they have been present in this genome for very long evolutionary times (the divergence values are so high that substitutions must be saturated, so that our times are under-estimated). Assuming a rate of change of 1.11 × 10^−8^ substitutions per site per year (see Methods), the RLs suggest that these satellites have been present on the sex chromosomes for at least 18 million years (my).

The examples shown in Fig. 3 suggest that, of the 6 satDNAs studied in detail, OocSat15-171, OocSat20-32, OocSat21-171, and OocSat35-28 have amplified differentially on the Y chromosome, generating loci with Y-specific clusters that are relatively young (divergence estimates that peak at about 10% or lower). The Y-specific peaks have sequence divergence around 4–8% corresponding to about 1.8–3.6 mya, though OocSat21-171 has a second peak with divergence near 22% (corresponding to 10 mya) and OocSat15-171 has several minor peaks with divergence around 25–32% (or about 11–14.4 mya). This is consistent with the FISH results (see above), with loci for these four satDNAs visible only on the Y chromosome. However, Fig. S6 shows that these four satDNA are also found on the X chromosome, albeit in much lower abundances, so they are not completely Y-specific. They are probably dispersed across the X chromosome as isolated units or short tandem arrays. Figure S6 suggests some local expansions (peaks at low divergence values) on the X (which would create small clusters of duplicated sequences undetectable by FISH) with divergence values from 4 to 44%, depending on the satDNA. Genome-wide total abundances of these four satDNAs are all higher in both males and females than those estimated from the isolated sex chromosomes (Tables S1 and S2), suggesting the presence of many clusters scattered throughout the autosomes. These autosomal clusters appear often to be old-established there, as their RL profiles in the sequences from male and female genome-wide samples (not shown) show peaks at higher divergences than those shown in Fig. 3, while those on the Y chromosome have amplified relatively recently.

OocSat47-35 and OocSat48-98 have much lower abundances than the other four examples shown (Table S1), but their RLs suggest differential amplification on the X, and, to a lesser extent, on the Y chromosome (these low abundance satDNA loci are not detectable by FISH, and we cannot exclude the possibility that they could be dispersed as short arrays throughout the Y). Despite its very low abundance, OocSat47-35 shows two conspicuous peaks (at 7% and 32% of sequence divergence, suggesting amplification at a much earlier period than the other amplification events), while the peaks for OocSat48-98 are at 4 and 14% divergence for the X copies (and slightly lower for the Y).

Table 1 shows divergence estimates for all satDNAs with repeat lengths of less than 151 bp obtained from the genomes of males and females, as well as between their sequences retrieved from the isolated X and Y sex chromosomes. The divergence between sex chromosome sequences ranged from 6 to 30%, depending on the satDNA (corresponding to between 2.7 and 13.5 my). Although these dates are rough, because differential amplification of individual sequence variants may have occurred on different chromosomes, sequences on the sex chromosomes have been diverging for longer than the autosomes and probably longer than the *O. octoguttata* species’ lifespan, consistent with recombination having stopped between the Y and X chromosomes in an ancestor. This is expected since the related species also have male achiasmy and giant sex chromosomes.

**Table 1:** Estimated pairwise divergence values of sequences of satellites shorter than 151 bp between the sexes (M-F) and within each sex (M = male, F = female) and divergence within and between sequences from the microdissected X and Y chromosomes (Y-X), or within sets of X or Y chromosome sequences. The divergence between sequences in the X and Y chromosomes was converted to divergence time, assuming 1.11 × 10^−8^ mutations per site per year (see Methods)

SatDNA family	Abundance (%)			Sex divergence			Sex chromosome divergence			Divergence time T = K/2 T (mya)	Chromosomal location
	Female	Male	M/F	M	F	M-F	Y	X	Y-X		
OocSat20-32	0.04	0.16	3.67	0.08	0.21	0.19	0.02	0.19	0.30	13.3	Y
OocSat24-20	0.07	0.14	1.95	0.10	0.11	0.11	0.03	na	na	na	Y + 24A
OocSat28-38	0.12	0.08	0.68	0.09	0.09	0.09	0.04	0.13	0.10	4.7	na
OocSat35-28	0.00	0.05	90.00	0.09	na	na	0.06	0.05	0.06	2.7	Y
OocSat41-92	0.02	0.02	1.00	0.17	0.18	0.18	0.22	0.21	0.22	10.0	na
OocSat42-30	0.01	0.01	1.75	0.07	0.05	0.07	0.01	0.00	0.01	0.4	2A
OocSat47-35	0.04	0.01	0.29	0.09	0.09	0.09	0.14	0.14	0.14	6.3	X
OocSat48-98	0.04	0.01	0.21	0.11	0.06	0.11	0.03	0.08	0.06	2.6	X
OocSat49-16	0.01	0.01	0.55	0.09	0.07	0.08	0.10	0.04	0.10	4.3	na

### Association of OocSatDNA with transposable elements (TEs)

For 49 OocSatDNA families, we asked whether the sequences resembled those of TEs. In 3 satDNAs belonging to the superfamily SF1 (OocSat10, OocSat14, and OocSat25), coverage (percentage of the sequence that matched with TEs) exceeded 50%, but others were lower (Table S4). Twenty-six satDNA families included sequences matching portions of TEs, with similarity percentages above 60%. Most matches were to Penelope; remarkably, this included all members of satellite superfamily SF1, though the percentage of these sequences matching the TE sequences was mostly (8 out of 11 satDNA families) below 50%. Other matches were to *Helitron* (satellite superfamily SF3) sequences and to DNA transposons (Table S4).

### Minimum spanning trees: MSTs

**Fig. 4:**
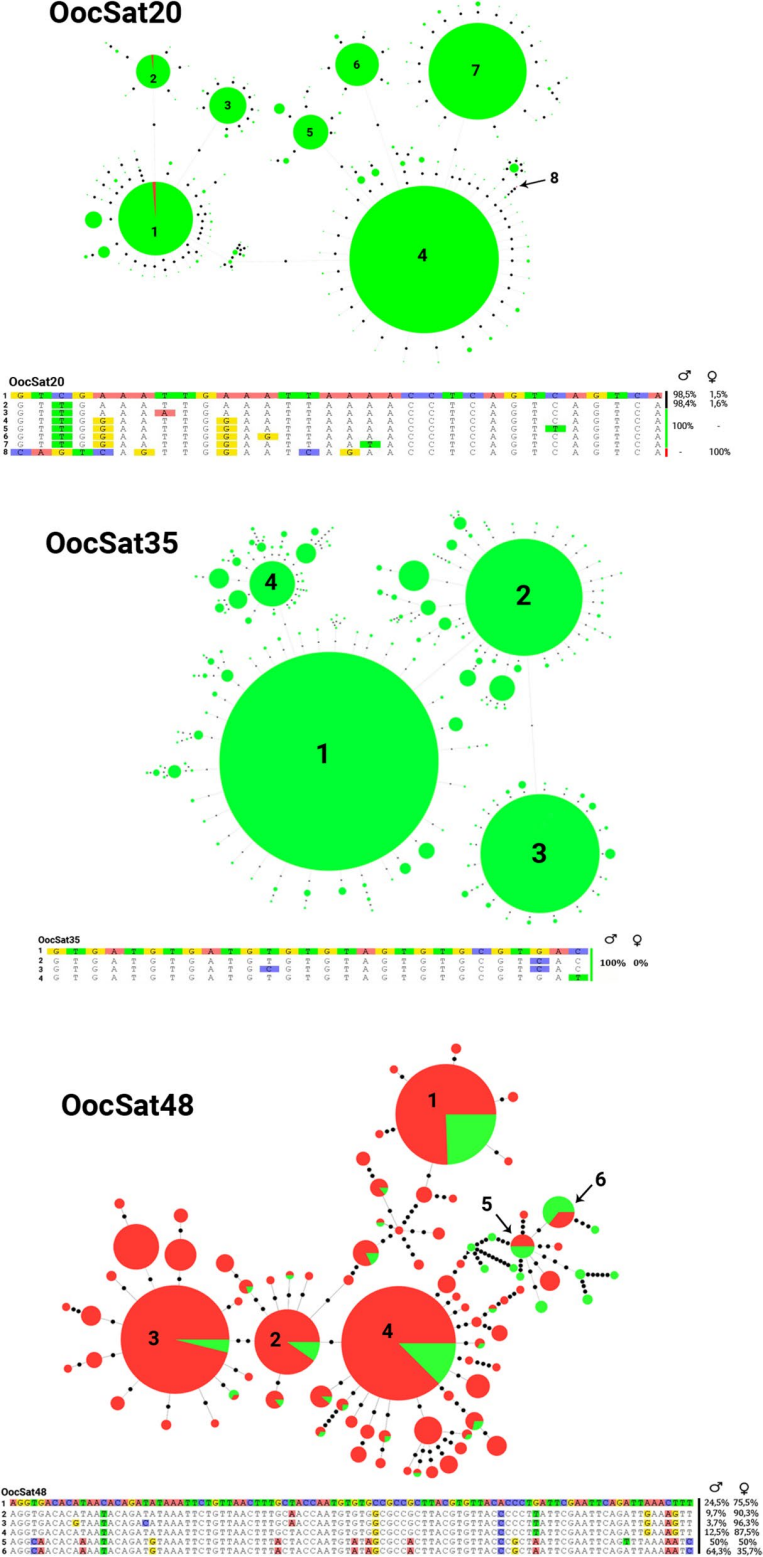
Linear minimum spanning trees (MSTs) of OocSat20, OocSat35, and OocSat48 obtained from reads from females (red) and males (green), showing variant haplotypes within these families. The numbers within the circles designate different major haplotypes of each of the three satDNA families, and the circle diameters are proportional to the representation of each haplotype. Black circles represent haplotypes differing by a single base pair from the neighboring haplotype

To investigate the extent of sharing between the sex chromosomes, we selected 3 satDNA families with different hybridization patterns in Figs. 1 and 2 and generated minimum spanning trees (MSTs) (Fig. 4). OocSat35 was not detected in females, but the MST of its sequences in males includes four haplotypes with diverse predominant monomers. Similarly, the MST of OocSat20 includes seven abundant haplotypes with diverse monomers in males and three haplotypes in females. The alignment of these haplotypes showed that those present in the male genome are all similar, but those in the female genome are variable. The results indicate Y-specific amplification and fixation of distinct sub-families that may have been present on both sex chromosomes, whereas the X chromosome retained a diverse set of OocSat20 repeats. In contrast, the MST of the OocSat48 family revealed abundant haplotypes shared between males and females, although some haplotypes are exclusive to females, in line with the FISH results (Figs. 1 and 2).

## Discussion

The *O. octoguttata* chromosomes are extremely puzzling, especially their exceptionally large X and Y chromosomes. At most, a single chromosome has fused with the X (see the Background and Fig. S5). Moreover, no fusion of two chromosomes with sizes like the autosomes of this or related species would create a new metacentric nearly as large as either the present X or Y. The X and Y must therefore have both undergone expansions. Their giant size (especially that of the X) is mysterious, and we discuss both in turn after first summarizing our findings concerning satDNAs.

### Organization of satDNAs in the *O. octoguttata* genome

A satellitome of 49 families (Table S1) is comparable with results from other insect species [14, 44, 49, 52, 59–63], and most OoSatDNAs were A + T-rich, as in other animals. The most significant difference from previously described satellitomes is their exceptional length (often > 1 kb) of most OocSatDNAs repeat units, including OocSat3, which is almost 5 kb (Table S1), even longer than the largest previously reported in Coleoptera that contains 3664 bp (CameSat120-3664), present in the genome of *Chrysolina americana* [49]; most are below 500 bp. No other property of the OocSatDNAs is extraordinary and suggests any reason for the large genome or the giant XY chromosome pair.

SatDNA abundances vary considerably among species, forming 51% of the large genome (2.9 Mb in the haploid genome) in one non-polyploid hemipteran insect [50]; however, the large genomes in some of these species may also have large TE contents [64]. The same probably applies to *O. octoguttata*, as the satDNA abundance is only about ~ 8–9%, less than in some previously analyzed beetle species [49, 61, 63]. As these sequences cannot explain its giant sex chromosomes, we can conclude that their expansion probably involved TE accumulation, as suggested by Rosolen et al. [37] and discussed further below.

Our results suggest that OocSatDNA amplification events have occurred in the past roughly 20 my. Many are found in long, high-copy number arrays in heterochromatic regions (Fig. 1), indicating tandem amplification. However, each OocSatDNA shows a different chromosomal distribution, which could reflect local tandem amplification or deletions, leading to arrays of various sizes [59]. As mentioned in the Results section, some OocSatDNAs showed no FISH signals and are probably present as small undetectable arrays dispersed in many genomic locations. Such a pattern would also be consistent with mobility involving TE activity. Our data cannot distinguish between these different possibilities.

### Achiasmatic male meiosis, the evolution and maintenance of huge Y chromosomes, and the evolution of sex-specific satDNAs

The large Y size, compared with the *O. octoguttata* autosomes, is consistent with achiasmy and evolution in the absence of recombination in males of the genus *Omophoita*. As explained in the Background, without crossing over, Y chromosome sequences will accumulate mutations and repetitive sequences (reviewed in [65]) independently of the X sequences (though gene conversion between different Y regions might occur). In principle, the giant Y chromosomes can therefore be understood as reflecting the amplification of repetitive sequences on this non-recombining chromosome (the X is discussed below). However, vast accumulation, creating a giant size, is highly unusual. We next discuss the possible evolution of the giant size of the X and Y chromosomes.

Assuming processes leading to tandem amplification in parts of both the Y and the X, independent amplification on each would create the observed different satDNA and TE contents of the X and Y chromosomes (Table 1 and Figs. 1 and 2, [37]). X- or Y-specific amplification events involving distinct repeat variants of each satDNA family appear to have occurred at different times (Figs. 3 and 4). However, the X and Y chromosomes share some satDNA sequences. It seems unlikely that sequences present in the sex chromosomes before recombination stopped became amplified differentially in one chromosome or the other, given the evidence that a non-recombining Y evolved when the giant sex chromosomes evolved in the mid-Cretaceous (see the Background). As many OocSatDNA sequences contain regions resembling those of TEs, we suggest that some may translocate between genomic regions, including the X and Y chromosomes, along with TE movements.

The Y and the X are also expected to evolve differently because of X recombination in females. Unlike the X, a long-established Y-linked region is therefore expected to undergo genetic degeneration (see the Background), which therefore seems likely to have occurred in these beetles. Y degeneration can help explain the larger size of the X than the Y chromosome, as reduced gene density on the Y, due to degeneration and to repeat accumulation, allows gene-poor areas to be deleted. Future sequencing should enable Y degeneration to be tested by identifying X-linked genes and estimating their coverage in males versus females. Even if a complete assembly of this repeat-rich genome is not possible, hemizygosity of X-linked genes in males (versus diploid coverage for autosomal and X-linked genes) can be detected in transcriptomes [66, 67].

If many genes have been lost from the Y, and it has become highly repetitive sequence content is expected to cause deletions, as has happened in Y and W chromosomes in most other groups or organisms (reviewed in [16]). A possible resolution of the puzzle that is consistent with our data is that repeated amplification of many different repeat types maintains these beetles’ extremely large Y chromosomes, or completely Y-linked regions (see the next section). A large X-linked region might be maintained similarly, as well as by the presence of functional genes.

### Pericentromeric regions

We next consider the Y expansion in more detail (the X is discussed below). If these beetles’ chromosomes have large, rarely recombining pericentromeric regions with high repeat densities, the evolution of a male-determining factor within such a region would create a large, completely (or almost completely) Y-linked region, as the Y chromosome is transmitted only through males (reviewed in [65]); such a region can potentially account for the Y’s expansion, as it would be expected to accumulate even more repetitive sequences than previously (though a giant size is nevertheless highly unusual). The OocSat15 family, exhibiting the greatest sex difference in abundance (Fig. S3; Tables S1 and S2), is indeed specifically accumulated in the Y chromosome pericentromeric region (Figs. 1 and 2), and many other repeat types have probably also contributed to the expansion. Genetic degeneration of the Y might permit repeated events involving rapid accumulation of repeats on this chromosome, unopposed by natural selection.

### The evolution and maintenance of huge X and Y chromosomes in the genus *Omophoita*

We concluded above that sex chromosome expansion probably involved TE accumulation, rather than satellite expansion, based on preliminary studies by Rosolen et al. [37] of the DNA (ClassII) transposon types, Tc1/Mariner-*Ooc*, found in most organisms so far studied [68]. Their FISH analyses indicated high accumulation on the sex chromosomes, but this was not quantified, and other TE types, such as the Penelope elements detected here (another DNA TE type, which dominates many invertebrate genomes [69] and is prone to “local hopping” events that may create tandem arrays [68], and/or satellites), may also contribute. In the plant *Rumex acetosa*, both TEs and satellites show Y accumulation. Importantly, however, the TE FISH signals were distributed very differently across the giant Y and X chromosomes of the three species examined [37]; the position differences cannot be explained by expansion of some repeats altering the positions of other large repeat arrays but suggest an intriguing process involving repeated replacement of expanded regions by new ones.

The huge size of the X is even more surprising than for a Y chromosome, as the X recombines in females, though recombination may be infrequent in the pericentromeric region. In *O. octoguttata*, one contribution to the larger X is probably simply that the Y chromosome has become smaller after degeneration, as just discussed. It is unlikely that the extraordinarily large size of this chromosome pair simply reflects this species’ very large genome. Although large satDNA content tends to correlate positively with genome size, this is not invariably the case; such a correlation is detected in the *Drosophila* subgenus, but not in *Sophophora* species [70]. Therefore, other factors must be involved.

Six OocSats had significantly higher abundances in the female than the male genome (Table S1) and two of them (named OocSat47 and OoCSat48) specifically map to the X chromosome (Figs. 1 and 2). Because our inferences are based on sequencing, not on FISH experiments alone, we can exclude one possible explanation for the X–Y difference: fragmentation of Y copies by insertion of other repeats into them [71]. More likely is a female-specific process creating extra X copies, as suggested for the *SlOgre1* TE of the Ty3/gypsy-like type detected in the plant *Silene latifolia*; other such situations are reviewed by Filatov et al. [72]. Again, however, the contributions of these elements cannot account quantitatively for the giant observed size of the X.

It is nevertheless worth asking whether the large X could reflect an expanded pericentromeric region. Arguing against this, neither the TE signals [37] nor the satDNAs studied here are concentrated at the centromeres of any of these species, which excludes simple expansions of the pericentromeric heterochromatin of the X and Y chromosomes.

*Omophoita* males, like male *Drosophila*, show male achiasmy, with very low recombination rates for all chromosome pairs. In such species, recombination rates for the X are the same as for the autosomes, since both recombine only in females (unlike species such as mammals, whose X recombines less than the autosomes, because the latter recombine in both sexes, not just in females). Therefore, the large size of the X in *Omophoita* species cannot be explained by a low recombination rate. However, the evolution of the X chromosome and the autosomes nevertheless differ, because, assuming a 1:1 sex ratio, the population includes only ¾ as many X chromosomes as autosomes (and only ¼ as many Ys as autosomes). The resulting lower X effective population size might be expected to allow the accumulation of repeats after an X-linked region evolves, assuming that insertions are not strongly disfavored by selection [73, 74]. A recent theoretical investigation indeed found that, for sex-linked regions that initially carry the same genes on both the Y and the X, so that X-linked loci are diploid, selection is expected (under a wide range of parameters) to be less effective than on autosomal or hemizygous X-linked loci [75]. These authors suggested that the reduced effective population size of such young X-linked regions may lead to changes like those predicted and observed in Y-linked regions, albeit to a much smaller extent; they specifically suggested that young X chromosomes may be liable to accumulate repeats. As discussed above, the *O. octoguttata* X chromosome appears to be ancient, not young, and its Y is probably strongly degenerated and may not carry alleles of most fully X-linked genes. The lower effective size of the X compared with the autosomes may nevertheless explain its higher accumulation of satDNAs and other repetitive DNA types than on other chromosomes.

Even in *Drosophila*, whose Ys are highly degenerated, the X chromosome euchromatin includes high proportions of satDNA sequences (1% in *D. melanogaster* and *D. simulans*, 2.4% in *D. mauritiana*, and at least 3.4% in *D. sechellia*), compared with only ∼0.07% for the autosomes [46]. In another beetle, *Tribolium castaneum*, the X chromosome satellite arrays are particularly long [76].

As local expansion is a major process for satDNA arrays [40], rarely recombining pericentromeric regions may be particularly prone to such expansions, as is observed in *Drosophila* [46, 77]. This may be especially likely if the pericentromeric region was already repeat-rich and had a low gene density, though this has not yet been formally modeled. It may also be particularly likely in species with extremely large genomes containing many sources that can potentially give rise to new repeats. Although the genome size of the beetle species studied here is large, much larger genomes are known in animals, including some amphibia and insects, with sizes considerably above 10 Gb [78], and TE accumulation is known to be involved, genome-wide TE content estimates can be as high as 75% [64]. TE silencing may also be less effective than in species with smaller genomes. Interestingly, these studies did not consistently detect correlations between the expression of TE silencing pathway genes and genome size or TE-derived PiRNA abundances. It is nevertheless possible that TE activity is high enough in *Omophoita* species to maintain high repetitive sequence densities, albeit with incessant turnover of the specific repeats present, as the results so far available [37] suggest (see above). Future, more comprehensive TE analyses will be valuable, including testing the suggestion that TEs may contribute to satDNA origin and amplification [45].

Sex-linked regions may also expand by the evolution of so-called ampliconic sequences of genes. These are palindrome structures often formed of closely spaced inverted repeats with very high identity, first detected in human genomes [78, 79], but also found in other sex chromosomes, including in the plant genus *Salix* [84]. Ampliconic gene families have been found on Y chromosomes in primates [79] and *Drosophila* [78, 79]. They can also lead to expansion of the X, and this may be especially likely in regions that recombine rarely because the resulting size difference between the X and Y will not lead to difficulties in meiosis if recombine occurs rarely. In the mouse, the X is estimated to include 19.4 Mb of ampliconic sequence, or about 12% of the 166 Mb chromosome; this includes 33 amplified genes and a mean of 11.4 copies of each of the more than 20 X-amplified genes with estimates [80–82] A neofunctionalized X-linked ampliconic gene family is essential for male fertility and equal sex ratio in mice [82]. Again, genome sequencing and analysis of coverage of genic sequences have the potential to test for such expansions in the future.

## Conclusions

Our findings define the major satDNA classes in flea beetle genomes and show that, in total, these form a minor portion of the total genome size. Although some are enriched on the Y, as predicted in a species with achiasmate males, they are not major components of the giant Ys. Moreover, unexpectedly, some are enriched on the X chromosome, though again, their amounts cannot account for the giant size of the X. The results suggest that different OocSatDNAs amplified independently at different times, as the limited data from TEs also suggest, though the TEs so far studied also cannot account for the giant sex chromosome sizes. It thus remains unclear whether certain major sequence types have contributed to the expansion of the Y and X chromosomes, or whether there is an overall tendency for Y and X expansion of many repetitive sequence types, and, if so, what promotes this. Obtaining a reliable reference genome assembly for this species will be difficult, given the high overall repetitive sequence content, especially for the giant X and Y chromosomes. Genome sequencing is not, however, essential. For example, comparisons of the locations of satellites in different species can also help suggest that repetitive sequences are constantly being replaced by others, as outlined above for the preliminary studies of TEs, which showed highly varied locations on the sex chromosomes of species with otherwise similar giant sex chromosomes.

## Methods

### Samples, chromosomal preparations, and DNA extraction

Twenty males and 10 females of *Omophoita octoguttata* were collected from the wild in Itaiacoca, PR, Brazil (25_07005.000 S 49_56025.300 W). All animals were collected with the authorization of the Brazilian environmental agency ICMBIO/SISBIO (license 15,402) and SISGEN (ABE8B7D). Meiotic chromosomes were examined following the protocol of [83]. Genomic DNAs (gDNAs) were extracted following [84], with modifications. The genomic DNA was extracted using the head, pronotum, and femur.

### Flow cytometry estimation of the *Omophoita octoguttata* genome size

A total of 11 individuals (5 males and 6 females) were processed for flow cytometric analysis according to the procedures described by [85], with some modifications. To avoid polyploid cells, we used head and leg tissue. No distinct peaks suggesting their presence were seen. *Astyanax* fish was used as a standard, as it is suitable for genome size estimating in other taxa, including invertebrate and vertebrate species [86–88].

Briefly, the entire head and legs of each individual were put in a 1.5-mL macrotube with 120 μL of lysis solution to obtain the nuclei. The samples were then incubated at room temperature (~ 27°C) for 20 min and vortexed every 5 min. To stain the nuclei, 1000 μL of Calcium-Free Dulbecco’s PBS (Sigma #D5773) containing 1 μg/mL of 4′,6-dimidino-2-phenylidole dihydrochloride (DAPI). Samples were filtered via a 50-μm mesh (Celltrics, Partec GmBH, Germany). The stained samples were then evaluated using a Partec CyFlow Plody Analyzer (Partec GmBH, Germany) with a filter set for DAPI excitation (358 nm). As a standard control, a small piece of tissue from yellowtail *Astyanax lacustris* (Lütken, 1875) (a characiform fish) was used, whose nuclear genome size was estimated previously to be 2.94 pg [89].

### Genome sequencing, satellitome characterization, and minimum spanning trees (MSTs)

Low-coverage shotgun sequences of genomic DNAs were obtained from one *O. octoguttata* male and one female. We also sequenced the DOP-PCR product amplified from each of fifteen microdissected X and Y chromosomes [31]. Due to their large size and asynapsis, these chromosomes appear isolated in nearly all metaphase plates (see Fig. 1), eliminating the risk of contamination during their isolation. As DOP-PCR preferentially amplifies repetitive elements (due to their high abundance), these libraries will include an overrepresentation of such sequences and can be used only for certain analyses (see below). Using the BGISEQ-500 platform at BGI (BGI Shenzhen Corporation, Shenzhen, China), these four libraries yielded 150 bp paired-end sequences, including 1 Gb for each of the female and male whole genomes.

Satellitome analysis was performed using the RepeatExplorer2 and Tandem Repeat Analyzer tools in the Galaxy platform [43, 90, 91], using default parameters, selecting Metazoa as the database in the REXdb, and comparative analysis with 2 groups (male and female), the SatMiner bioinformatic tool described by Ruiz-Ruano et al. [44] (https://gitlab.com/fjruizruano/satminer/-/blob/master/README.md). The satellite DNA sequences are available on GenBank-NCBI under the accession numbers PP188098–PP188146.

SatDNAs were characterized for both the male and female genome sequences and separately for the X and Y chromosome sequences. After FastQC analysis of the sequence reads, we used Trimmomatic [92] to discard low-quality reads with Q < 20, using the options LEADING:3 TRAILING:3 SLIDINGWINDOW:4:20 MINLEN: 100 CROP: 101, as described by Ruiz-Ruano et al. [44]. We then followed the procedure recommended by Ruiz-Ruano et al. [44]. After removing low-quality reads, we randomly selected 200.000 forward and the same number of reverse reads from our male and female individuals, using the SeqTK software (https://github.com/lh3/seqtk) [93], and concatenated the reads from each sex, adding the suffix XY or XX to denote the sequenced male and female, respectively. These concatenated reads were analyzed using RepeatExplorer2 (https://repeatexplorer-elixir.cerit-sc.cz/galaxy). Specifically, to yield a satDNA database for the species, putative satDNA sequences were filtered from the raw reads using DeconSeq [94] until no further putative satDNAs were found.

To remove any sequences with similarities with multigene families before performing homology searches, we filtered the likely satDNA sequences using the software rm_homology (https://github.com/fjruizruano/satminer/blob/master/rm_homology.py). To analyze similarity among putative satDNAs identified by the TAREAN software [43], including matches between them, we aligned each satDNA against the entire set of putative satDNAs using the Cross_match search engine of RepeatMasker [95]. Sequences in the groups thus identified were aligned and sets with nucleotide identity < 80% were classified as belonging to different superfamilies, as suggested by Ruiz-Ruano et al. [44]. The resulting satDNA families were numbered in order of decreasing abundance in the male genome. The two output files from the RepeatExplorer pipeline (named CLUSTER_TABLE.csv and COMPARATIVE_ANALYSIS_COUNTS.csv) were used to run the script “plot comparative clustering summary.R” to generate a comparative visualization of repetitive element results (Fig. S3).

To generate a consensus monomer sequence for each satDNA cluster of thousands of copies, we used TAREAN [43]. Each individual repeat is diverged from the consensus sequence, and these differences are termed sequence variants. To estimate the abundance and divergence to the consensus of each sequence variant, we aligned a sample of 8 million randomly selected reads to their consensus sequences, using RepeatMasker and the script at https://github.com/fjruizruano/satminer/blob/master/repeat_masker_run_big.py, and used RepeatMasker’s calcDivergenceFromAlign.pl tool to obtain a histogram of the genomic proportions plotted against the Kimura 2-parameter divergence from the consensus for each sequence variant. Such plots are termed “repeat landscapes” or RLs [95] which displays, for each satDNA family, the abundance of the different repeat variants (Y-axis) and their percentage of sequence divergence from its consensus sequence (X-axis). We also obtained RLs for the sex chromosome sequences in a similar way, using a total of 14 million reads randomly selected from the microdissected X and Y chromosome amplified DNA. To estimate rough divergence times of each satDNA sequence from its consensus sequence, we transformed the divergence estimates in the RLs using T = K/2r, where r = 1.11 × 10^−8^ nucleotide changes per site per year (one generation per year), the mean turnover rate estimated from the analysis of the grasshopper satellitome [59]. Remarkably, the mean turnover rate estimated from the analysis of the grasshopper satellitome gives us a similar value to that estimated for other plant sex-chromosomes’ satDNAs [96]. satDNAs arrays one of the most rapidly evolving parts of the genome [48] and this rate of change is higher than the average mutation rate estimated for most vertebrates and invertebrates [97–99].

### General repeat composition of the *O. octoguttata* genome

To get an initial idea of the repeat composition of the *O. octoguttata* male and female genomes, we first ran RepeatModeler2 [100] on the assembled genome of another Alticinae beetle species, named *Crepidodera aurea* (NCBI access number GCA_949320105.2). This is an automated pipeline for genomic discovery of transposable element families, which first builds a database from sequences from the genome of interest, then runs RepeatModeler2 to classify the sequences into known types. Our analysis (using the RepeatModeler2 default parameters) was used to create an Alticinae beetle repetitive sequence database, using 500,000 paired-end reads from *O. octoguttata*. Then, to estimate the genomic proportions of each repetitive element type (satDNAs and transposable elements) in the *O. octoguttata* reads, which sample the genome as a whole, we ran RepeatMasker using the custom python script (https://github.com/fjruizruano/satminer/blob/master/repeat_masker_run_big.py) mentioned above. The results were plotted as a pie chart, with all satDNA families classified as a single “satellite DNA” class.

### Evolutionary analysis of satDNAs

The development time of *O. octoguttata* individuals from egg to adult takes approximately 50 days. However, breeding is seasonal, with a population peak in the summer, and declining in the winter [101]. We therefore assumed one, or at most two, generations per year.

To estimate the abundances of individual satDNAs accurately and to obtain information about monomer diversity, each male and female satDNA was used as a reference to extract monomers from the genomic libraries for both sexes. We chose three satellites with monomer lengths below 151 bp, OocSat20 and OoSat35 (which, in our FISH experiments described below mapped exclusively on the Y chromosome) and OocSat48 (which mapped exclusively on the X). To do this, we subsampled 2 sets of 4,000,000 paired reads from each genomic library (see above), aligned them with dimer sequences of the three satDNAs using Bowtie2, and then removed the sequence corresponding to one monomer, following [102]. Next, we used CD-HIT to eliminate sequences that appear only once (singletons) which may result from sequencing errors (as recommended by Fu et al. [103]). We also aligned the sets of sequences of each of these three satDNAs independently with MUSCLE [104] and eliminated monomers that were found only once before creating minimum spanning trees (MSTs) for each of the three satDNAs using PHYLOVIZ [105].

In addition to sequences of OocSat20, OoSat35, and OocSat48, we also extracted repeat units of several satellites (OocSat20, OocSat24, OocSat28, OocSat35, OocSat41, OocSat47, OocSat48, and OocSat49) with monomer lengths < 151 bp (sometimes much less than this) that were found in the individual X and Y chromosome’ libraries. We aligned the sequences with Clustal X [106] and calculated mean genetic distances using the program MEGA v.11 [107]. Calculations included male–female comparisons and comparisons between sequences isolated from the sex chromosomes. We transformed to time these divergence values to numbers of years as above. Finally, each satDNA was subjected to BLAST-searches [108] in NCBI to check for previously described sequences. Also, to search for other transposable elements, we searched Repbase [109] for homologies with transposable elements using CENSOR [110], with the “no_low” and “no_is” options, using “Arthropoda” as the sequence source.

### Fluorescence in situ hybridization, including primer design and polymerase chain reaction

For 16 of the 49 OocSatDNAs characterized, we designed primers for PCR amplifications to make probes for in situ hybridization experiments (Table S5). The OocSatDNAs chosen included (i) the five most abundant satDNAs, (ii) the nine satDNAs with the greatest differences in abundance between the sexes, and (iii) two satellites that were associated with Penelope TE sequences (see the Results section and Table S4). The PCR reactions used an initial denaturation step of 95°C for 5 min, followed by 32 cycles with 95°C for 20 s, with 50–55°C as annealing for 30 s, and 72°C for 40 s, and a final extension step of 72°C for 5 min. Electrophoresis on 2% agarose gels confirmed amplification of the specific satDNAs, based on their expected sizes. Finally, the products were quantified using the NanoDrop spectrophotometer (ThermoFisher Scientific, Branchburg, NJ, USA).

Probes were made from the satDNAs PCR products and were labeled with Atto550-dUTP (Jena Biosciences, Jena, Germany). Five OocSatDNAs with repeat unit lengths < 40 bp (OocSat20, OocSat24, OocSat35, OocSat42, and OocSat47) were directly labeled with biotin or Cy3 at the 5′ end during synthesis, which was carried out by ThermoFisher Scientific (Waltham, MA, USA).

Telomeric sequences (TTAGG)n were also generated by PCR using the (TTAGG)5 and (CCTAA)3 primers [111], in the absence of a DNA template, following [112] and were directly labeled with Atto550-dUTP by Nick-Translation (Jena Biosciences, Jena, Germany), following the manufacturer instructions.

Probe mixtures for in situ hybridizations included 200 ng of labeled satDNA plus 50% of formamide, 2ΧSSC, 10% SDS, 10% dextran sulfate, and Denhardt’s buffer at pH 7.0 in a total volume of 20 µL. The FISH experiments were performed under high-stringency conditions [113]. The slides were dehydrated in ethanol (70%, 85%, and 100%), before counterstaining chromosomes with DAPI mounted in Vectashield (Vector Laboratories, Burlingame, USA). Eleven out of the 16 OocSatDNAs selected for FISH investigations showed positive hybridization signals.

## Supplementary Information

Supplementary Material 1.

Supplementary Material 2.

## Data Availability

The datasets generated during and/or analyzed during the current study are available in the NCBI database (https://www.ncbi.nlm.nih.gov/bioproject/) under accession numbers PP188098 - PP188146, and in the SRA-NCBI for the Omophoita octoguttata male (SRX23444021), female (SRX23444022), microdissected X chromosomes (SRX23445951), and microdissected Y chromosomes (SRX23445952). All other data generated or analyzed during this study are included in this published article and its supplementary information files.

## Abbreviations

satDNA: Satellite DNA
OocSatDNAs: Satellite DNAs of *Omophoita octoguttata*
TEs: Transposable elements
MST: Minimum spanning trees
SF: Superfamily
RUL: Repeat unit length
FISH: Fluorescence in situ hybridization
PCR: Polymerase chain reaction
DAPI: 4′,6-Diamidino-2-phenylindole
My: Million years
ICMBIO: Chico Mendes Institute of Biodiversity Conservation
SISBIO: Brazilian Biodiversity Information and Authorization System
SISGEN: National System of Genetic Resource Management and Associated Traditional Knowledge
BR: Brazil
