# Supplementary material for: Unraveling the role of satellite DNAs in the evolution of the giant XY sex chromosomes of the flea beetle Omophoita octoguttata (Coleoptera, Chrysomelidae)
Source: BMC Biol. 2025 Feb 21;23:53. doi: 10.1186/s12915-025-02155-5 (PMC11846391; doi:10.1186/s12915-025-02155-5)
Supplement: Supplementary file 1 — Supplementary Material 1. [file 12915_2025_2155_MOESM1_ESM.docx]

**Table S1:** General features of *Omophoita octoguttata* satellitome*.* The OocSatDNA selected for FISH experiments are highlighted in bold.

| **SatDNA family** | **RUL** | **A+T** | **Abundance (F)** | **Abundance (M)** | **Abundance (M/F)** | **Divergence(M)** | **Divergence (F)** | **SF** |
| --- | --- | --- | --- | --- | --- | --- | --- | --- |
| OocSat01 - 183 | 183 | 66% | 0.01995 | 0.02027 | 1.01597 | 10.77 | 10.72 | 2 |
| OocSat02 - 392 | 392 | 64% | 0.00529 | 0.00619 | 1.17155 | 5.54 | 5.54 |  |
| OocSat03 - 1275 | 1275 | 69% | 0.00482 | 0.00452 | 0.93783 | 6.16 | 6.1 |  |
| OocSat04 - 2658 | 2658 | 64% | 0.00388 | 0.00423 | 1.09105 | 13.24 | 13.12 | 1 |
| OocSat05 - 399 | 399 | 63% | 0.00273 | 0.00337 | 1.23591 | 7.49 | 8.85 |  |
| OocSat06 - 738 | 738 | 67% | 0.00290 | 0.00323 | 1.11328 | 5.75 | 6.17 | 3 |
| OocSat07 - 431 | 431 | 62% | 0.00293 | 0.00320 | 1.09197 | 11.03 | 12 |  |
| OocSat08 - 2653 | 2653 | 64% | 0.00231 | 0.00269 | 1.16452 | 13.65 | 15.16 | 1 |
| OocSat09 - 148 | 148 | 66% | 0.00453 | 0.00256 | 0.56603 | 6.72 | 7.64 |  |
| OocSat10 - 2714 | 2714 | 70% | 0.00211 | 0.00253 | 1.19656 | 4.37 | 4.49 | 1 |
| OocSat11 - 465 | 465 | 58% | 0.00261 | 0.00250 | 0.95634 | 17.42 | 17.39 |  |
| OocSat12 - 2785 | 2785 | 70% | 0.00209 | 0.00239 | 1.13998 | 7.57 | 8.13 | 1 |
| OocSat13 - 2654 | 2654 | 65% | 0.00198 | 0.00235 | 1.18527 | 12.07 | 12.34 | 1 |
| OocSat14 - 2768 | 2768 | 65% | 0.00147 | 0.00229 | 1.55078 | 8.08 | 9.96 | 1 |
| OocSat15 - 171 | 171 | 67% | 0.00009 | 0.00210 | 24.35021 | 9.98 | 35.94 |  |
| OocSat16 - 144 | 144 | 65% | 0.00150 | 0.00203 | 1.35630 | 3.53 | 3.7 |  |
| OocSat17 - 2813 | 2813 | 71% | 0.00183 | 0.00204 | 1.11422 | 10.63 | 11.28 | 1 |
| OocSat18 - 2577 | 2577 | 65% | 0.00169 | 0.00199 | 1.17311 | 21.91 | 22.38 | 1 |
| OocSat19 - 826 | 826 | 66% | 0.00181 | 0.00162 | 0.89726 | 5 | 4.95 | 3 |
| OocSat20 - 32 | 32 | 63% | 0.00043 | 0.00158 | 3.67039 | 9.41 | 20.76 |  |
| OocSat21 - 171 | 171 | 66% | 0.00001 | 0.00150 | 114.89617 | 10.28 | 19.33 |  |
| OocSat22 - 2663 | 2663 | 67% | 0.00113 | 0.00145 | 1.28375 | 13.25 | 14.04 |  |
| OocSat23 - 155 | 155 | 73% | 0.00127 | 0.00143 | 1.12344 | 1.37 | 1.43 |  |
| OocSat24 - 20 | 20 | 65% | 0.00073 | 0.00142 | 1.94017 | 8.51 | 8.51 |  |
| OocSat25 - 2693 | 2693 | 72% | 0.00136 | 0.00131 | 0.96257 | 3.69 | 3.82 | 1 |
| OocSat26 - 2728 | 2728 | 75% | 0.00093 | 0.00121 | 1.30743 | 7.77 | 9.1 | 1 |
| OocSat27 - 156 | 156 | 60% | 0.00125 | 0.00107 | 0.85731 | 6.81 | 6.72 |  |
| OocSat28 - 38 | 38 | 61% | 0.00121 | 0.00082 | 0.68162 | 7.02 | 6.9 |  |
| OocSat29 - 302 | 302 | 63% | 0.00053 | 0.00072 | 1.35225 | 12.98 | 10.93 |  |
| OocSat30 - 2466 | 2466 | 66% | 0.00066 | 0.00071 | 1.07493 | 18.54 | 17.88 | 1 |
| OocSat31 - 5042 | 5042 | 67% | 0.00087 | 0.00068 | 0.78092 | 6.78 | 7.47 |  |
| OocSat32 - 3225 | 3225 | 62% | 0.00078 | 0.00067 | 0.85416 | 6.19 | 6.13 |  |
| OocSat33 - 318 | 318 | 67% | 0.00060 | 0.00062 | 1.02886 | 2.84 | 2.77 |  |
| OocSat34 - 160 | 160 | 66% | 0.00073 | 0.00060 | 0.81720 | 13.93 | 13.45 | 2 |
| OocSat35 - 28 | 28 | 50% | 0.00001 | 0.00045 | 89.17505 | 5.35 | 13.52 |  |
| OocSat36 - 1325 | 1325 | 63% | 0.00077 | 0.00043 | 0.56522 | 7.77 | 6.17 |  |
| OocSat37 - 1848 | 1848 | 62% | 0.00046 | 0.00031 | 0.67352 | 2.55 | 2.38 |  |
| OocSat38 - 1306 | 1306 | 63% | 0.00036 | 0.00026 | 0.71190 | 27.39 | 25.17 |  |
| OocSat39 - 203 | 203 | 58% | 0.00016 | 0.00020 | 1.23689 | 5.32 | 3.99 |  |
| OocSat40 - 126 | 126 | 62% | 0.00008 | 0.00016 | 2.04024 | 7.61 | 7.22 |  |
| OocSat41 - 92 | 92 | 63% | 0.00016 | 0.00016 | 1.02553 | 15.35 | 15.54 |  |
| OocSat42 - 30 | 30 | 57% | 0.00008 | 0.00014 | 1.77218 | 5.63 | 4.61 |  |
| OocSat43 - 562 | 562 | 51% | 0.00025 | 0.00013 | 0.53436 | 13.55 | 12.14 |  |
| OocSat44 - 746 | 746 | 71% | 0.00010 | 0.00013 | 1.30730 | 2.79 | 2.12 |  |
| OocSat45 - 127 | 127 | 66% | 0.00011 | 0.00011 | 0.99211 | 5.89 | 6.05 |  |
| OocSat46 - 333 | 333 | 66% | 0.00016 | 0.00010 | 0.62169 | 6.77 | 6.2 |  |
| OocSat47 - 35 | 35 | 46% | 0.00035 | 0.00010 | 0.28340 | 14.72 | 11.18 |  |
| OocSat48 - 98 | 98 | 61% | 0.00039 | 0.00008 | 0.21075 | 10.25 | 4.99 |  |
| OocSat49 - 16 | 16 | 69% | 0.00011 | 0.00006 | 0.56161 | 6.16 | 6.07 |  |

**Table S2:** General features of *Omophoita octoguttata* satellitome of X and Y chromosomes.

|  | **Abundance** | | | **Divergence** | |
| --- | --- | --- | --- | --- | --- |
| **SatDNA family** | **Y** | **X** | **Y/X** | **Y** | **X** |
| **OocSat01 - 183** | 0.000113 | 0.000049 | 2.331074 | 21.38 | 14.23 |
| **OocSat02 - 392** | 0.003512 | 0.003028 | 1.159795 | 6.58 | 6.64 |
| OocSat03 - 1275 | 0.001874 | 0.002764 | 0.678138 | 7.25 | 7.10 |
| OocSat04 - 2658 | 0.002218 | 0.001371 | 1.618556 | 14.10 | 14.76 |
| **OocSat05 - 399** | 0.009521 | 0.004176 | 2.279822 | 4.64 | 6.89 |
| OocSat06 - 738 | 0.001009 | 0.000761 | 1.325371 | 8.93 | 9.00 |
| OocSat07 - 431 | 0.001867 | 0.001562 | 1.195769 | 9.88 | 13.44 |
| OocSat08 - 2653 | 0.001429 | 0.000932 | 1.532155 | 10.56 | 14.24 |
| OocSat09 - 148 | 0.000119 | 0.000417 | 0.284708 | 11.38 | 10.52 |
| OocSat10 - 2714 | 0.001427 | 0.001265 | 1.128623 | 5.21 | 5.01 |
| OocSat11 - 465 | 0.000730 | 0.000673 | 1.084898 | 14.98 | 16.57 |
| OocSat12 - 2785 | 0.004339 | 0.003649 | 1.189224 | 5.30 | 5.92 |
| OocSat13 - 2654 | 0.001601 | 0.001097 | 1.460479 | 9.56 | 11.45 |
| OocSat14 - 2768 | 0.004507 | 0.001637 | 2.753806 | 5.39 | 8.48 |
| **OocSat15 - 171** | 0.001515 | 0.000003 | 471.112115 | 8.19 | 22.13 |
| OocSat16 - 144 | 0.000001 | 0.000003 | 0.307692 | 6.86 | 8.44 |
| OocSat17 - 2813 | 0.002564 | 0.001704 | 1.504721 | 9.27 | 9.70 |
| OocSat18 - 2577 | 0.001132 | 0.000838 | 1.351655 | 18.37 | 19.69 |
| OocSat19 - 826 | 0.000571 | 0.000980 | 0.582304 | 5.39 | 5.32 |
| **OocSat20 - 32** | 0.014746 | 0.000473 | 31.184986 | 5.92 | 18.94 |
| **OocSat21 - 171** | 0.001418 | 0.000001 | 2210.514477 | 11.70 | 11.87 |
| OocSat22 - 2663 | 0.000725 | 0.000402 | 1.803853 | 15.44 | 19.58 |
| OocSat23 - 155 | 0.000018 | 0.000034 | 0.526278 | 2.45 | 2.64 |
| **OocSat24 - 20** | 0.000048 | 0.000000 | 988.098039 | 8.66 | 22.46 |
| OocSat25 - 2693 | 0.000922 | 0.001264 | 0.729514 | 3.90 | 3.86 |
| OocSat26 - 2728 | 0.000371 | 0.000367 | 1.009266 | 6.41 | 10.91 |
| OocSat27 - 156 | 0.000054 | 0.000047 | 1.149357 | 8.87 | 9.25 |
| OocSat28 - 38 | 0.000006 | 0.000026 | 0.214103 | 10.04 | 9.62 |
| OocSat29 - 302 | 0.000019 | 0.000005 | 3.978174 | 18.06 | 6.30 |
| OocSat30 - 2466 | 0.000340 | 0.000308 | 1.104859 | 16.86 | 15.18 |
| OocSat31 - 5042 | 0.000449 | 0.000915 | 0.490324 | 7.90 | 7.78 |
| OocSat32 - 3225 | 0.000276 | 0.000440 | 0.626772 | 6.54 | 7.46 |
| OocSat33 - 318 | 0.000003 | 0.000010 | 0.263296 | 4.34 | 5.77 |
| OocSat34 - 160 | 0.000093 | 0.000090 | 1.037694 | 16.57 | 14.35 |
| **OocSat35 - 28** | 0.017995 | 0.000007 | 2676.320963 | 6.48 | 8.25 |
| OocSat36 - 1325 | 0.000220 | 0.000629 | 0.349255 | 6.64 | 6.81 |
| OocSat37 - 1848 | 0.000500 | 0.002331 | 0.214503 | 2.48 | 2.29 |
| OocSat38 - 1306 | 0.000085 | 0.000330 | 0.256856 | 18.98 | 13.17 |
| OocSat39 - 203 | 0.000083 | 0.000009 | 9.675576 | 17.96 | 4.94 |
| **OocSat40 - 126** | 0.000001 | 0.000000 | ----------- | 22.73 | ------- |
| OocSat41 - 92 | 0.000076 | 0.000157 | 0.479968 | 13.59 | 14.10 |
| **OocSat42 - 30** | 0.000725 | 0.000001 | 917.487349 | 4.84 | 8.65 |
| OocSat43 - 562 | 0.000065 | 0.000168 | 0.384281 | 11.32 | 10.65 |
| OocSat44 - 746 | 0.000064 | 0.000217 | 0.296176 | 3.43 | 3.15 |
| OocSat45 - 127 | 0.000345 | 0.000003 | 128.819459 | 5.96 | 15.51 |
| OocSat46 - 333 | 0.000048 | 0.000093 | 0.512565 | 5.77 | 6.45 |
| **OocSat47 - 35** | 0.000194 | 0.000638 | 0.303414 | 14.33 | 14.64 |
| **OocSat48 - 98** | 0.000011 | 0.000035 | 0.324994 | 8.45 | 12.06 |
| OocSat49 - 16 | 0.000050 | 0.000186 | 0.269982 | 9.16 | 8.19 |
| **Total** | 0.079998 | 0.036094 |  |  |  |

**Table S3.** Distribution and abundance of OocSatDNAs on the *O. octogutatta* sex chromosomes. The table also indicates when a satellite type is found on autosomes, and the number of autosomes (indicated by A) on which such satellites were detected. ch - centromeric heterochromatin; h = heterochromatin and e = euchromatin.

| **SatDNAs** | **Chromosomal Location** | **Abundance** | | |
| --- | --- | --- | --- | --- |
|  |  | **Y** | **X** | **X/Y** |
| OocSat01 - 183 | 20A (ch) | 0.000113 | 0.000049 | 2.331074122 |
| OocSat02 - 392 | X + Y + 20A (ch, h, e) | 0.003512 | 0.003028 | 1.159794565 |
| OocSat05 - 399 | X + Y + 20A (h, e) | 0.009521 | 0.004176 | 2.279822279 |
| OocSat15 - 171 | Y (ch) | 0.001515 | 0.000003 | 471.1121149 |
| OocSat20 - 32 | Y (h) | 0.014746 | 0.000473 | 31.18498601 |
| OocSat21 - 171 | Y (h) | 0.001418 | 0.000001 | 2210.514477 |
| OocSat24 - 20 | Y + 20A (h) | 0.000048 | 0.000000 | 988.0980392 |
| OocSat35 - 28 | Y (h) | 0.017995 | 0.000007 | 2676.320963 |
| OocSat42 - 30 | 2A (ch) | 0.000001 | ----------- | ------------ |
| OocSat47 - 35 | X (h) | 0.000725 | 0.000001 | 917.4873494 |
| OocSat48 - 98 | X (ch, h) | 0.000011 | 0.000035 | 0.32499352 |

**Table S4:** Homology of satDNAs with transposable elements. Different columns indicate the following data: **From/To** contains the beginning/ending of the positions of the fragment on the query sequence. Name: name of the matching repeat; Class: : class/subclass of matching repeat as specified in repeat annotation; Dir indicates the orientation ('d' for direct, 'c' for complementary) of the repeat fragment; Sim contains the value of similarity between 2 aligned fragments; Score: this column contains the alignment score obtained from blast Coverage, percentage of matching between the query sequence (satDNA sequence) and the matching repeat; Total Coverage, sum of the partial percentages of matching between the query sequence (satDNA sequence) and the matching repeat.

| Satellite DNA family | From | To | Name | Class | Dir | Sim | Score | Coverage (%) | Total (%) |
| --- | --- | --- | --- | --- | --- | --- | --- | --- | --- |
| OocSat01-183 | 67 | 119 | Academ-8_LMi | DNA/Academ | c | 0.7736 | 235 | 28,42 | 28,42 |
| OocSat03-1275 | 67 | 103 | Helitron-3_FoCa | DNA/Helitron | c | 0.8649 | 246 | 2,82 |  |
| OocSat03-1275 | 367 | 447 | Gypsy-15_DEl-I | LTR/Gypsy | d | 0.7349 | 209 | 6,27 |  |
| OocSat03-1275 | 803 | 879 | RTE-1_RhyDom | NonLTR/RTE | d | 0.8158 | 252 | 5,96 | 15,06 |
| OocSat04-2658 | 534 | 1491 | Penelope-12_LVa | NonLTR/Penelope/Poseidon | d | 0.6808 | 1387 | 36,00 |  |
| OocSat04-2658 | 1900 | 1952 | Penelope-1_ChOp | NonLTR/Penelope/Poseidon | d | 0.7736 | 249 | 1,96 | 37,96 |
| OocSat06-738 | 15 | 72 | MINIME_DN | Interspersed_Repeat | d | 0.8448 | 413 | 7,72 |  |
| OocSat06-738 | 125 | 384 | Helitron-1_FoCa | DNA/Helitron | d | 0.6899 | 699 | 35,09 |  |
| OocSat06-738 | 468 | 613 | CR1-6_ChOp | NonLTR/CR1 | d | 0.7324 | 313 | 19,65 | 62,47 |
| OocSat08-2653 | 33 | 91 | Gypsy-16_DePu-I | LTR/Gypsy | d | 0.7869 | 224 | 2,19 |  |
| OocSat08-2653 | 665 | 718 | Penelope-8_LVa | NonLTR/Penelope/Poseidon | c | 0.7963 | 305 | 2,00 |  |
| OocSat08-2653 | 1093 | 1847 | Penelope-7_LVa | NonLTR/Penelope/Poseidon | c | 0.6702 | 1112 | 28,42 |  |
| OocSat08-2653 | 1930 | 2005 | EnSpm-2N1_DSuz | DNA/EnSpm/CACTA | c | 0.7595 | 237 | 2,83 | 35,43 |
| OocSat10-2714 | 32 | 70 | DNA-45B_LSal | DNA | d | 0.8684 | 227 | 1,40 |  |
| OocSat10-2714 | 318 | 2092 | Penelope-8_SiOr | NonLTR/Penelope/Poseidon | c | 0.6554 | 1399 | 65,36 |  |
| OocSat10-2714 | 2106 | 2663 | Penelope-5_SiOr | NonLTR/Penelope/Poseidon | c | 0.6518 | 305 | 20,52 | 87,29 |
| OocSat12-2785 | 773 | 845 | Kiri-21_AAe | NonLTR/Kiri | d | 0.7467 | 220 | 2,59 |  |
| OocSat12-2785 | 925 | 977 | Penelope-9_PMon | NonLTR/Penelope | d | 0.7736 | 241 | 1,87 |  |
| OocSat12-2785 | 1125 | 1183 | Penelope-13_SiOr | NonLTR/Penelope/Poseidon | d | 0.7627 | 238 | 2,08 |  |
| OocSat12-2785 | 1272 | 1630 | Penelope-85_LMi | NonLTR/Penelope/Poseidon | d | 0.6886 | 447 | 12,85 |  |
| OocSat12-2785 | 1679 | 1741 | Penelope-1_SiOr | NonLTR/Penelope/Poseidon | d | 0.7705 | 209 | 2,23 |  |
| OocSat12-2785 | 1819 | 1933 | Gypsy-9_SogFur-I | LTR/Gypsy | c | 0.6786 | 243 | 4,09 |  |
| OocSat12-2785 | 2027 | 2092 | Penelope-11_CoFl | NonLTR/Penelope/Poseidon | d | 0.7576 | 296 | 2,33 |  |
| OocSat12-2785 | 2236 | 2276 | hAT-1_PBa | DNA/hAT | c | 0.8333 | 203 | 1,44 |  |
| OocSat12-2785 | 2377 | 2452 | Gypsy-365_AA-I | LTR/Gypsy | d | 0.7733 | 239 | 2,69 | 32,17 |
| OocSat13-2654 | 641 | 740 | Penelope-3_ChOp | NonLTR/Penelope/Poseidon | c | 0.6600 | 297 | 3,73 |  |
| OocSat13-2654 | 1121 | 2052 | Penelope-12_LVa | NonLTR/Penelope/Poseidon | c | 0.6659 | 1107 | 35,08 | 38,81 |
| OocSat14-2768 | 357 | 427 | Penelope-1_LMi | NonLTR/Penelope/Poseidon | d | 0.7746 | 311 | 2,53 |  |
| OocSat14-2768 | 539 | 618 | Penelope-3_FoCa | NonLTR/Penelope/Poseidon | d | 0.7500 | 285 | 2,85 |  |
| OocSat14-2768 | 625 | 2074 | Penelope-3_SiOr | NonLTR/Penelope/Poseidon | d | 0.6823 | 2458 | 52,35 | 57,73 |
| OocSat17-2813 | 582 | 664 | Penelope-7_TrVa | NonLTR/Penelope/Poseidon | c | 0.7284 | 268 | 2,92 |  |
| OocSat17-2813 | 836 | 1611 | Penelope-13_SiOr | NonLTR/Penelope/Poseidon | d | 0.6578 | 853 | 27,55 |  |
| OocSat17-2813 | 1677 | 1757 | MuDR-3_LSal | DNA/MuDR | c | 0.7590 | 214 | 2,84 |  |
| OocSat17-2813 | 1848 | 2013 | Penelope-11_CoFl | NonLTR/Penelope/Poseidon | d | 0.6726 | 239 | 5,87 |  |
| OocSat17-2813 | 2207 | 2252 | TransibN1_DP | DNA/Transib | d | 0.7872 | 220 | 1,60 | 40,77 |
| OocSat18-2577 | 413 | 499 | Penelope-7N1_PMon | NonLTR/Penelope | c | 0.7273 | 277 | 3,34 |  |
| OocSat18-2577 | 904 | 1641 | Penelope-12_LVa | NonLTR/Penelope/Poseidon | c | 0.6635 | 1038 | 28,60 |  |
| OocSat18-2577 | 1664 | 1699 | DNAV-1d_LVa | IntegratedVirus/DNAV | c | 0.8649 | 207 | 1,36 |  |
| OocSat18-2577 | 2369 | 2425 | BEL-84_AntGra-I | LTR/BEL | c | 0.7586 | 217 | 2,17 | 35,47 |
| OocSat19-826 | 392 | 556 | Helitron-1_FoCa | DNA/Helitron | d | 0.6848 | 416 | 19,85 |  |
| OocSat19-826 | 674 | 758 | Gypsy-7_CydSpl-I | LTR/Gypsy | d | 0.7857 | 252 | 10,17 | 30,02 |
| OocSat22-2663 | 706 | 1656 | Penelope-12_LVa | NonLTR/Penelope/Poseidon | d | 0.6607 | 1017 | 35,67 |  |
| OocSat22-2663 | 2045 | 2285 | Penelope-11_LVa | NonLTR/Penelope/Poseidon | d | 0.7186 | 387 | 9,01 | 44,69 |
| OocSat25-2693 | 200 | 496 | Penelope-85_LMi | NonLTR/Penelope/Poseidon | d | 0.7244 | 360 | 10,99 |  |
| OocSat25-2693 | 632 | 2239 | Penelope-2_SiOr | NonLTR/Penelope/Poseidon | d | 0.6873 | 1417 | 59,67 |  |
| OocSat25-2693 | 2564 | 2605 | DNA8-4_PBa | DNA | d | 0.8333 | 200 | 1,52 | 72,19 |
| OocSat26-2728 | 441 | 554 | BEL-9_SiOr-I | LTR/BEL | c | 0.7653 | 250 | 4,14 |  |
| OocSat26-2728 | 610 | 713 | Penelope-1_SiOr | NonLTR/Penelope/Poseidon | c | 0.7629 | 264 | 3,78 |  |
| OocSat26-2728 | 1017 | 1250 | Penelope-1_RhyDom | NonLTR/Penelope/Poseidon | c | 0.6725 | 269 | 8,54 |  |
| OocSat26-2728 | 1311 | 1708 | Penelope-3_SiOr | NonLTR/Penelope/Poseidon | c | 0.6813 | 349 | 14,55 |  |
| OocSat26-2728 | 1762 | 2104 | Penelope-4_BTa | NonLTR/Penelope/Poseidon | c | 0.6960 | 436 | 12,54 |  |
| OocSat26-2728 | 2140 | 2268 | DNA-84_LSal | DNA | c | 0.7500 | 224 | 4,69 |  |
| OocSat26-2728 | 2389 | 2496 | Gypsy-31_DAlb-LTR | LTR/Gypsy | c | 0.7624 | 260 | 3,92 | 52,16 |
| OocSat29-302 | 252 | 295 | Copia-27_DPu-I | LTR/Copia | d | 0.7727 | 239 | 14,24 | 14,24 |
| OocSat30-2466 | 416 | 519 | Penelope-3_ChOp | NonLTR/Penelope/Poseidon | c | 0.6442 | 239 | 4,18 |  |
| OocSat30-2466 | 1248 | 1318 | Penelope-1_ChOp | NonLTR/Penelope/Poseidon | c | 0.7465 | 316 | 2,84 |  |
| OocSat30-2466 | 1633 | 2443 | Penelope-7_PMon | NonLTR/Penelope/Poseidon | c | 0.6704 | 1326 | 32,85 | 39,86 |
| OocSat31-5042 | 1102 | 1335 | Gypsy-19_DAlb-I | LTR/Gypsy | d | 0.6889 | 295 | 4,62 |  |
| OocSat31-5042 | 1741 | 1822 | R1-9_BTa | NonLTR/R1 | d | 0.7500 | 222 | 1,61 |  |
| OocSat31-5042 | 2275 | 2321 | Gypsy-4_CQ-I | LTR/Gypsy | d | 0.8367 | 219 | 0,91 |  |
| OocSat31-5042 | 2692 | 2724 | Gypsy-12_Hm | LTR | c | 0.8788 | 251 | 0,63 |  |
| OocSat31-5042 | 4184 | 4228 | TART_DV | NonLTR/Jockey | d | 0.7778 | 251 | 0,87 |  |
| OocSat31-5042 | 4518 | 4573 | BEL-19_AntGra-I | LTR/BEL | d | 0.7818 | 223 | 1,09 |  |
| OocSat31-5042 | 4657 | 4716 | Gypsy-20_DAlb-I | LTR/Gypsy | d | 0.8525 | 262 | 1,17 | 10,91 |
| OocSat32-3225 | 254 | 373 | Polinton-1_SiMi | DNA/Polinton | d | 0.7460 | 226 | 3,69 |  |
| OocSat32-3225 | 1017 | 1185 | Gypsy-6_SiMi-I | LTR/Gypsy | c | 0.6647 | 226 | 5,21 |  |
| OocSat32-3225 | 1207 | 1239 | Gypsy-18_PlaPen-I | LTR/Gypsy | c | 0.8485 | 225 | 0,99 |  |
| OocSat32-3225 | 1716 | 1767 | R2Amel | NonLTR/R2 | c | 0.7547 | 221 | 1,58 |  |
| OocSat32-3225 | 2064 | 2126 | Gypsy-4_DBp-I | LTR/Gypsy | c | 0.8382 | 329 | 1,92 |  |
| OocSat32-3225 | 2898 | 2966 | Gypsy-48_ChOp-I | LTR/Gypsy | c | 0.7910 | 262 | 2,11 |  |
| OocSat32-3225 | 3036 | 3085 | BEL-56_HedSal-I | LTR/BEL | c | 0.7600 | 243 | 1,52 | 17,02 |
| OocSat33-318 | 228 | 274 | Polinton-3_DSuz | DNA/Polinton | d | 0.8125 | 208 | 14,47 | 14,47 |
| OocSat34-160 | 45 | 105 | Academ-N1_RPr | DNA/Academ | c | 0.8095 | 250 | 37,50 | 37,50 |
| OocSat36-1325 | 8 | 147 | Mariner-6_DSuz | DNA/Mariner | d | 0.8156 | 761 | 10,49 | 10,49 |
| OocSat37-1848 | 956 | 1011 | Cynin-2_PH | NonLTR | c | 0.7895 | 249 | 2,98 |  |
| OocSat37-1848 | 1608 | 1651 | Gypsy-5_DMo-I | LTR/Gypsy | c | 0.8182 | 208 | 2,33 | 5,30 |
| OocSat38-1306 | 229 | 307 | Gypsy-16_RhyDom-I | LTR/Gypsy | c | 0.7027 | 214 | 5,97 |  |
| OocSat38-1306 | 1000 | 1047 | hAT-39_DWil | DNA/hAT | d | 0.7755 | 204 | 3,60 | 9,57 |
| OocSat43-562 | 100 | 244 | Gypsy-1B_DSuz-I | LTR/Gypsy | c | 0.6853 | 234 | 25,62 | 25,62 |
| OocSat44-746 | 62 | 146 | BEL-630_AA-I | LTR/BEL | d | 0.7619 | 262 | 11,26 |  |
| OocSat44-746 | 500 | 568 | Gypsy-121_PMon-LTR | LTR/Gypsy | c | 0.7571 | 212 | 9,12 |  |
| OocSat44-746 | 578 | 677 | BEL-8_NeoCor-I | LTR/BEL | c | 0.7822 | 286 | 13,27 | 33,65 |

**Table S5:** Primers designed for the OocSatDNA found in the genome of *Omophoita octoguttata.*

| **SatDNA Family** | **Primer Foward** | **Primer Reverse** |
| --- | --- | --- |
| OocSat01-183 | AATACTGGAGAGCTGGAG | AGTTAGGGAGCTGTGACA |
| OocSat02-392 | ACGTAATGGTGCAGTGCT | ATTCCGAGATCCACCGAT |
| OocSat03-1275 | TAATTACGCAGTGTTGCGTT | ATAACGCCCTTTGGTCCT |
| OocSat04-2658 | TCCTATTACATCGTGCTTATTC | AATATGACCTTCTCCGACCA |
| OocSat05-399 | ATTACCCGACCAATTAGTTC | ATCCTGTGTGAACCGAAC |
| OocSat13-2654 | ATTGAGTCTACATAAGGCAA | TCCAACTATTGACCTCCG |
| OocSat15-171 | CACGTTTTACAGGGCATTT | ACGTGAAGAGTGCAAGCT |
| OocSat18-2577 | CTGAACTTCTCCCTATCG | TACCGTTCTGAAGGATATAG |
| OocSat20-32 | AAACCTCAGTCAGTCAGTTGGAATTGGAATTA | Biotin labbeling |
| OocSat21-171 | CAATAAGCTACAGTTGAATATC | AAGCTTATGTGTCATACAAATG |
| OocSat24-20 | AGAATTGTCTCGAATTGTCT | Biotin labbeling |
| OocSat35-28 | ATGTGTGTAGTGTGCGTGACGTGATGTG | Cy3 - labbeling |
| OocSat40-126 | CGATGTTGAGAATTTAGTCCTC | GTTGAGCCTCTGTGGTCGTC |
| OocSat42-30 | CAAATCGGGATAAACTCTTCCGTTCTTCCA | Biotin labbeling |
| OocSat47-35 | GTTAGATAGGCGAGTTGGATGCGAAGCACCCGACT | Biotin labbeling |
| OocSat48-98 | CTGTTAACTTTGCTACCAAT | ATATCTGTATTATGTGTCACCT |
