# Supplementary material for: Unraveling the role of satellite DNAs in the evolution of the giant XY sex chromosomes of the flea beetle Omophoita octoguttata (Coleoptera, Chrysomelidae)
Source: BMC Biol. 2025 Feb 21;23:53. doi: 10.1186/s12915-025-02155-5 (PMC11846391; doi:10.1186/s12915-025-02155-5)
Supplement: Supplementary file 2 — Supplementary Material 2. [file 12915_2025_2155_MOESM2_ESM.docx]

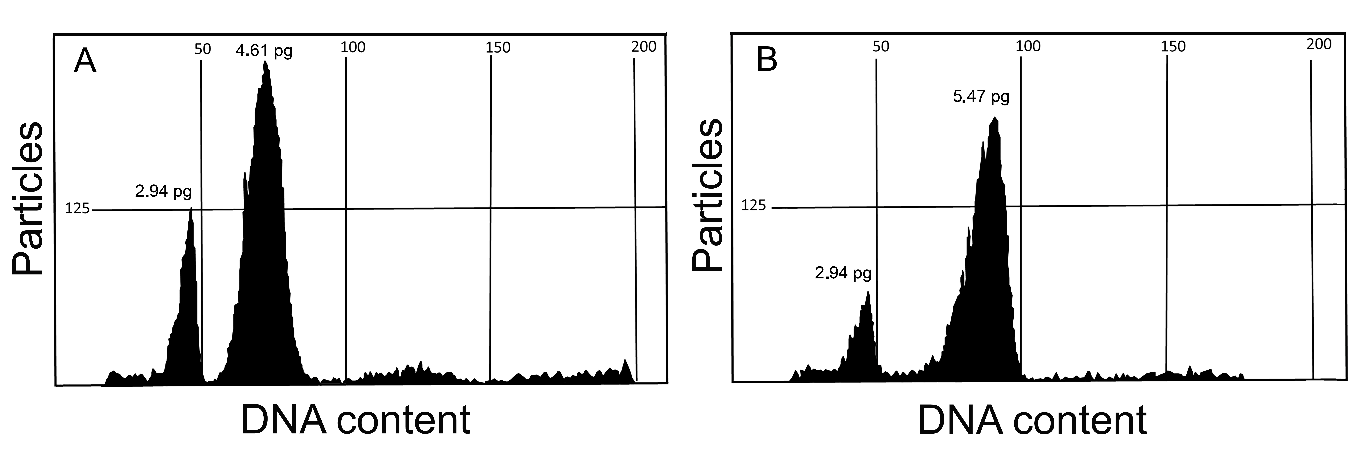


**Figure S1.** Flow cytometric charts depict the genome size of *Omophoita octoguttata*. The first peak on A and B is the genome size (2n) of *Astyanax lacustris*. The major peak on A is the genome size of males, while the major peak on B is the genome size of females of *O. octoguttata*.


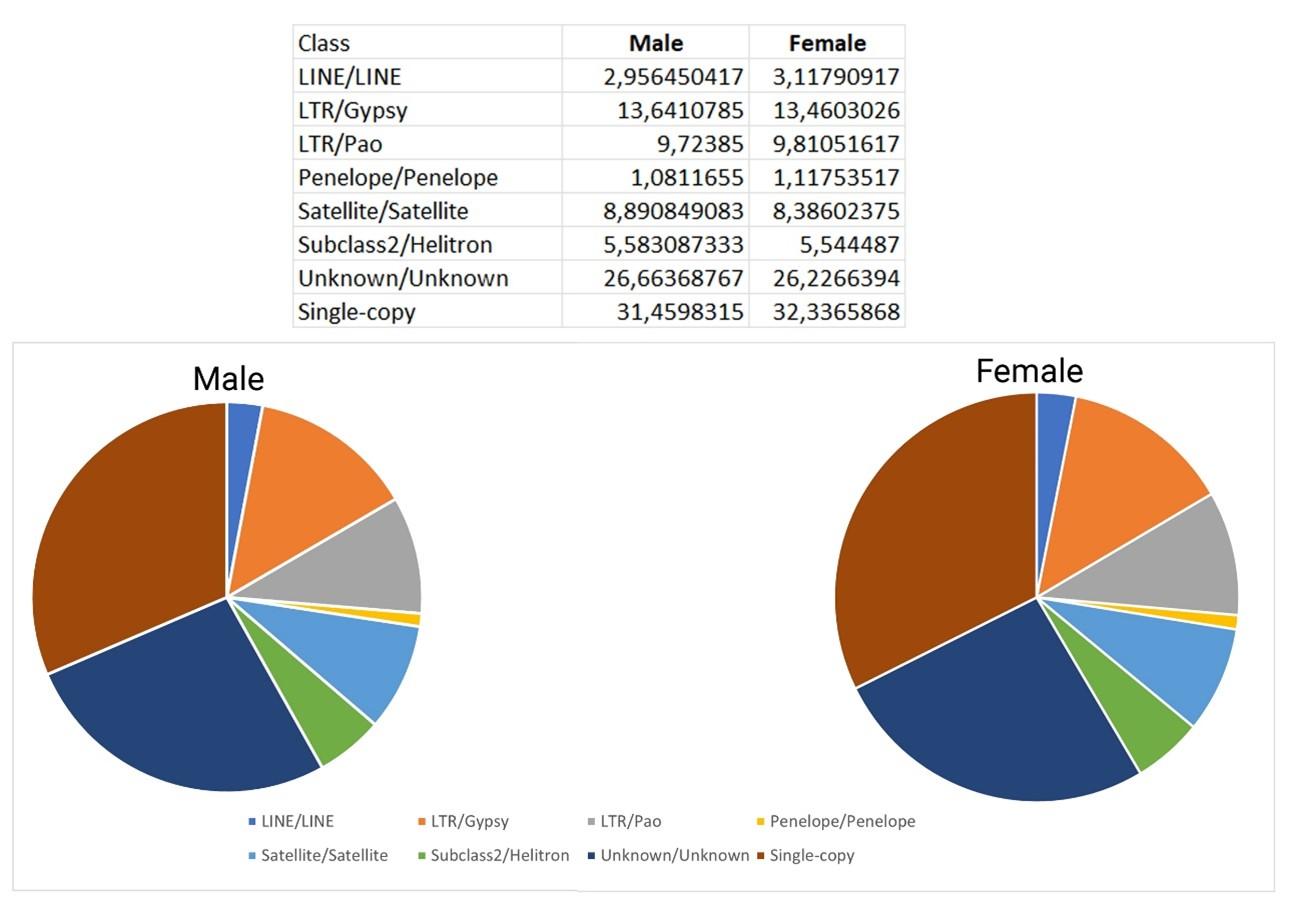


**Figure S2.** Summary of repeatome analysis in males and females from *O. octoguttata.*


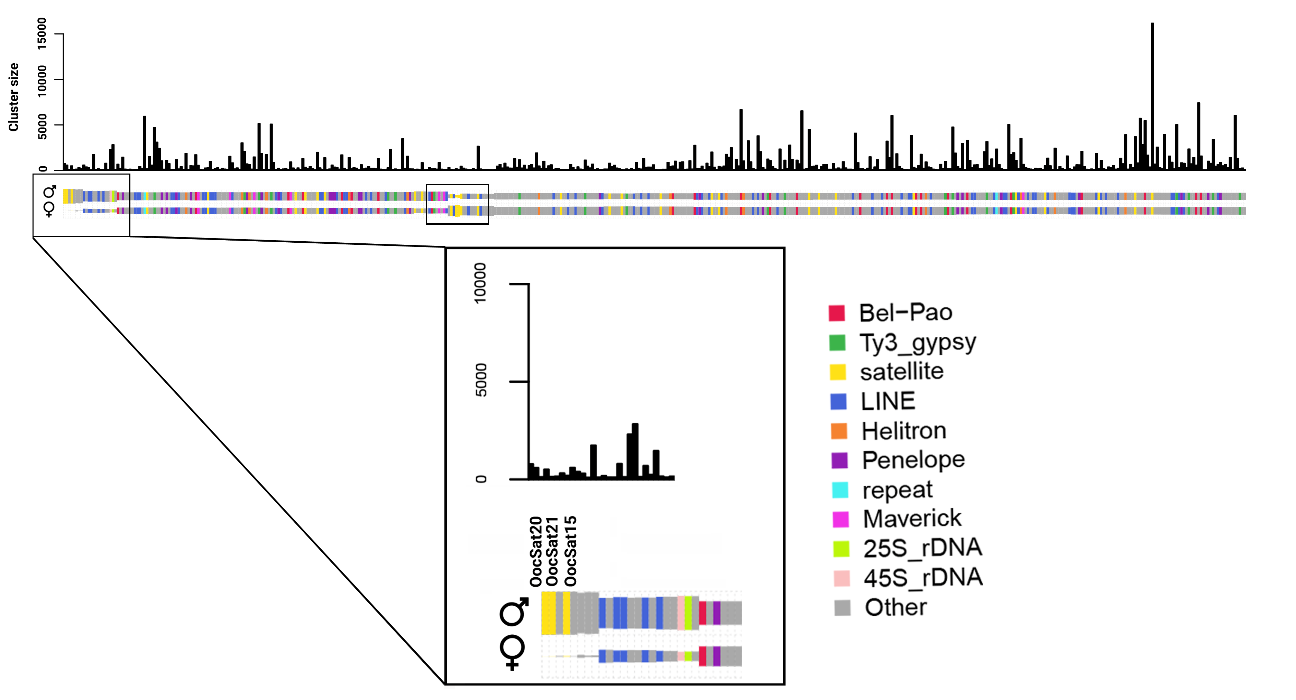


**Figure S3.** Comparison between the results of hierarchical clustering to sort the samples and clusters of repetitive DNA from the male (XY) and female (XX) of *O. octogutatta*. The upper bar plot shows the total number of reads for each repeat type cluster. Among others, there are very striking differences in repeat composition between males and females related to three specific satDNA families (named OocSat15, OocSat20, and OocSat21) that show the largest difference in their abundance between males and females **(Table S2).**

**
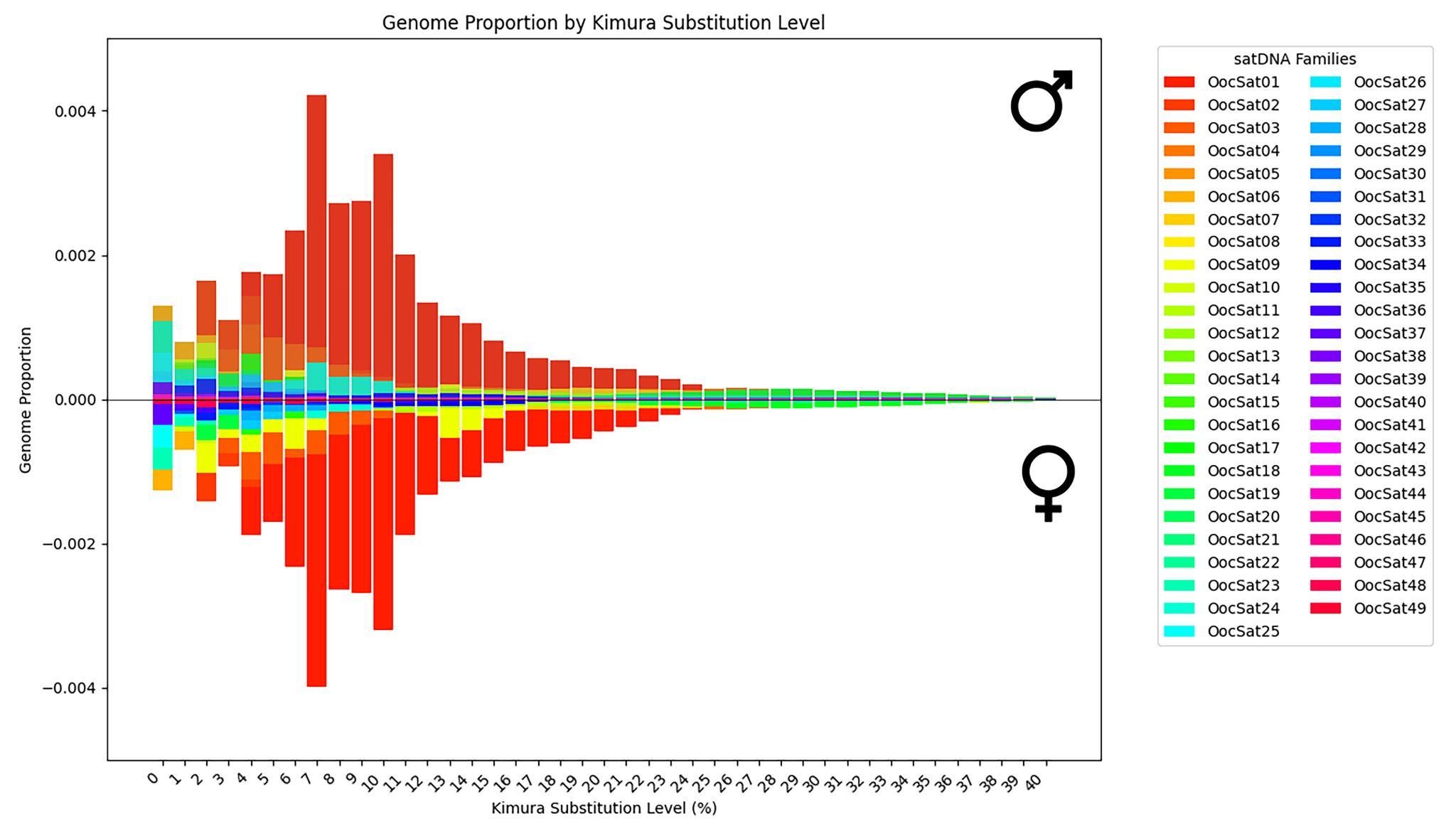
**

**Figure S4.** Repeat landscape showing the genome abundance and divergence (Kimura substitution level) of OocSatDNAs identified on male and female genomes.


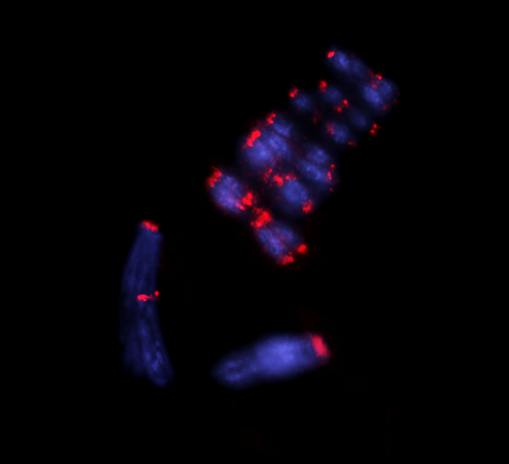


**Figure S5.** Male meiotic chromosomes of *O. octoguttata* showing the chromosomal locations of telomere probes (TTAGG). The arrowhead indicates the interstitial telomeric site on the long arm of the X chromosome. Bar = 20 µm.


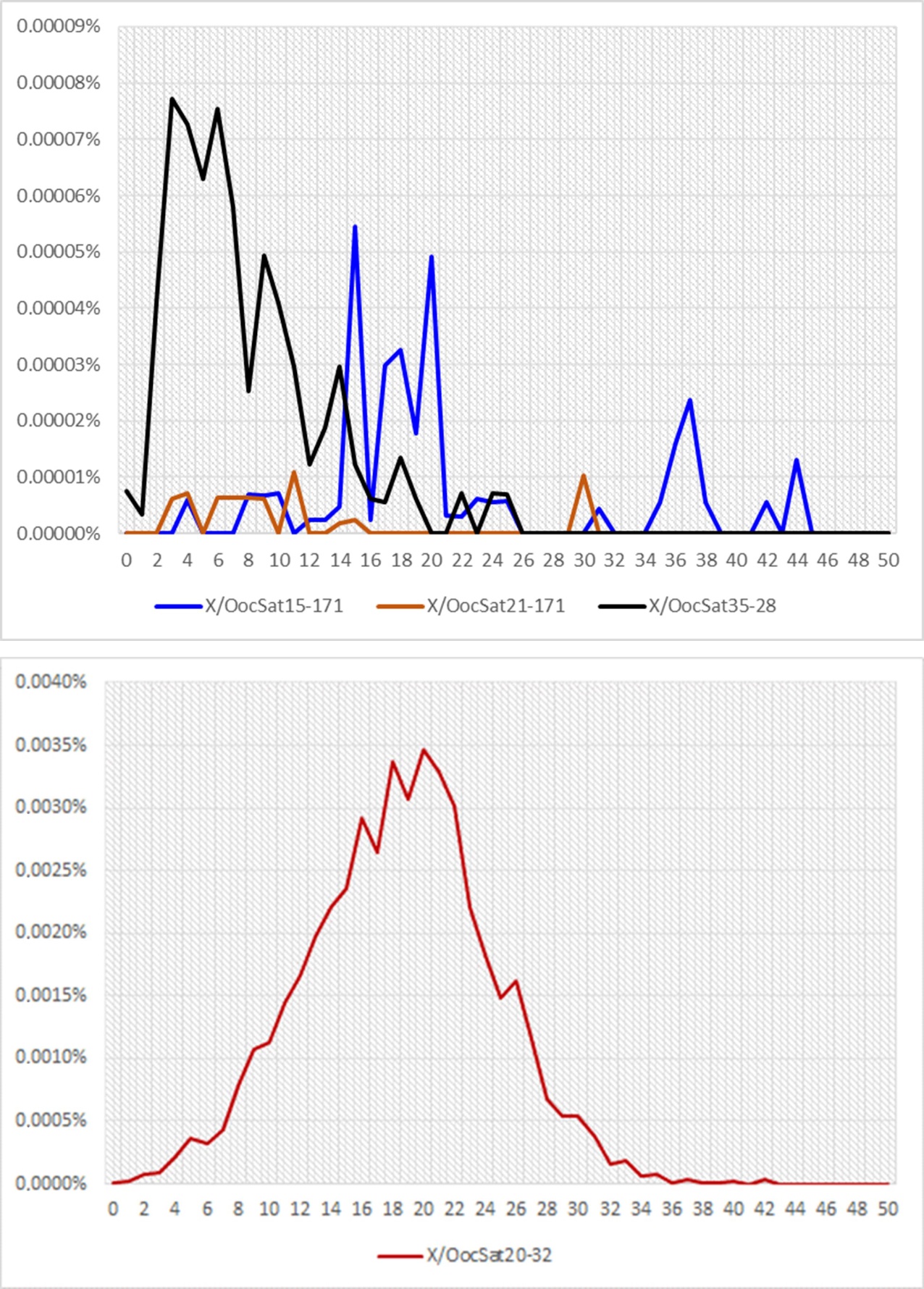


**Figure S6.** RLs plots of the sequences of different satDNAs (OocSat15-171, OocSat20-32, OocSat21-171 and OocSat35-28) rescaled to observe minimal array expansions occurring on the X chromosome.
